# A fourth subgenome of the *Camelina* genus reveals gene dominance is influenced by chromosomal proximity

**DOI:** 10.1038/s41467-026-74800-8

**Published:** 2026-06-26

**Authors:** Raju Chaudhary, Kevin C. Koh, Peng Gao, Sampath Perumal, Erin E. Higgins, Kyla Horner, Stephen J. Robinson, Zhengping Wang, Christina Eynck, Venkat Bandi, Andrew G. Sharpe, Isobel A. P. Parkin

**Affiliations:** 1 Global Institute for Food Security, University of Saskatchewan, Saskatoon, SK Canada; 2 Agriculture and Agri-Food Canada, Saskatoon, SK Canada; 3 National Research Council of Canada, Saskatoon, SK Canada

**Keywords:** Polyploidy in plants, Genome evolution, Agricultural genetics, Evolutionary genetics

## Abstract

*Camelina sativa*, an oilseed crop of the Brassicaceae family, has close relatives that vary in ploidy levels, providing a unique platform for studying plant genome evolution. Here, we report an improved assembly of the widely used *C. sativa* reference DH55 and three additional genome assemblies of *Camelina microcarpa*: one tetraploid, and two hexaploids with divergent chromosome numbers, Type 1 (2n = 40) and Type 2 (2n = 38). We uncover the fourth subgenome of the *Camelina* genus that of *C. microcarpa* Type 2, which shows numerous unique chromosomal rearrangements differentiating it from other characterized *Camelina* subgenomes. In this recently formed species, the second subgenome displays gene expression dominance, contrary to expectations from the two-step evolutionary process invoked in the generation of related Brassicaceae species. The observed gene dominance is negatively correlated with inter-subgenome chromatin interaction frequencies, suggesting chromosome conformation and proximity in the nucleus contribute to this mechanism of genome evolution.

## Abstract (web summary)

The *Camelina* genus comprises diploid, tetraploid, and hexaploid species. Here, the authors complete genome assembly of ploidy series by assembling genomes of a tetraploid and two hexaploid *C. microcarpa* accessions, along with the cultivated hexaploid *C. sativa*, and reveal chromosomal proximity–driven gene dominance.

## Introduction

*Camelina sativa* (L.) Crantz is a hexaploid species of the Brassicaceae family^1^. Due to a unique fatty acid composition as well as protein content, *C. sativa* has shown economic potential as an ingredient for the aquaculture, poultry feed and cosmetics industries, as well as being favored for human consumption^2^. One factor limiting crop improvement in *C. sativa* has been the low natural genetic diversity within the species^3–5^.

In other crops, related or wild species have shown potential as sources of novel variation^6^; in this context, identification of diverse haplotypes for regions of interest in related *Camelina* species, coupled with suitable genomic resources, might be useful in furthering work in *C. sativa*^7^. Several wild relatives of *C. sativa* have been reported, with some well-defined taxonomically, while others show evidence of cryptic species with comparable morphologies^8^. *Camelina microcarpa* Andrz. ex DC is one of the closest wild relatives of this crop and was believed to encompass three levels of ploidy: diploid, tetraploid and hexaploid^9^. For the latter level, two different hexaploid *C. microcarpa* species with distinct combinations of three subgenomes have been now identified^10^. The first hexaploid *C. microcarpa* appears to share three subgenomes with *C. sativa* (hereafter *C. microcarpa* Type 1 or CmiT1), whereas the second, *C. microcarpa* (hereafter *C. microcarpa* Type 2 or CmiT2), shares only two subgenomes with *C. sativa*, while the third subgenome of this species is distinct from any of the three subgenomes of *C. sativa*^10^. A number of whole genome duplication events and chromosomal rearrangements seem to have occurred in the generation of the higher ploidy *Camelina* species. The characterization of the subgenome composition in *Camelina* species will provide a strategy for interspecific hybridization in *C. sativa* and could potentially help in broadening the narrow genetic base of *C. sativa* germplasm^3,4,9^.

After whole-genome hybridisation in an allopolyploid, the orthologous duplicate genes commonly show evidence of higher gene expression for a particular subgenome (genome dominance) and gene loss (fractionation), which is generally more prevalent in the less dominant subgenome. Such observations led to the two-step hypothesis in the formation of Brassica paleo-hexaploids^11^, whereby the addition of each subgenome led to inferred dominance over the resident genome, generating a hierarchy of gene loss from the Least Fractionated (LF) to the Most Fractionated (MF) subgenomes^12^. Although gene fractionation is relatively low in *C. sativa*^13^, likely due to its recent emergence as a polyploid, there are marked differences in gene expression among the orthologs and the third subgenome was generally found to be dominant^10,14^. The exact mechanism controlling subgenome gene expression dominance is not known, although chromosomal rearrangements such as homoeologous exchanges or differences in chromosome architecture, such as the level of transposons, have both been associated with the observed effect on gene expression^15–17^. Likewise, epigenetic and structural changes after genome merger could also play a role in shaping gene expression in the polyploids^18,19^. However, there are species which have either undergone limited or unbiased fractionation after genome duplication, such as *Cucurbita* and soybean, respectively^20,21^. For *C. microcarpa* T2, the lack of the third dominant subgenome of *C. sativa* presents an interesting candidate to explore the nature of subgenome dominance. Further, the availability of genome assemblies for diploid *Camelina neglecta*^22^ and diploid *Camelina hispida* (Boiss.) Hedge^23^, both posited as representative progenitors for *C. sativa*, provides an opportunity to explore the evolution of genome structure in higher ploidy *Camelina* species.

Here, we sequence the genome of tetraploid *C. microcarpa* (CN119243) (2n = 26), hexaploid *C. microcarpa* T1 (CN119205) (2n = 40), hexaploid *C. microcarpa* T2 (accession CN120025) (2n = 38) and hexaploid *C. sativa* (accession DH55). We reveal the subgenome structure of a fourth subgenome for the *Camelina* genus. The relationship among subgenomes is explored in terms of age of divergence, syntenic genomic block rearrangements, methylation status, transposon distribution and chromatin interactions. Gene expression dominance is observed in all three hexaploid *Camelina* species, although is more pronounced in *C. microcarpa* T2, where the non-dominant genomes show higher numbers of chromosomal interactions, suggesting reduced proximity of the dominant genome allows independent and hence greater gene expression.

## Results

### De novo genome assembly, repeat analysis, and gene annotation of *Camelina* genomes

**Fig. 1:**
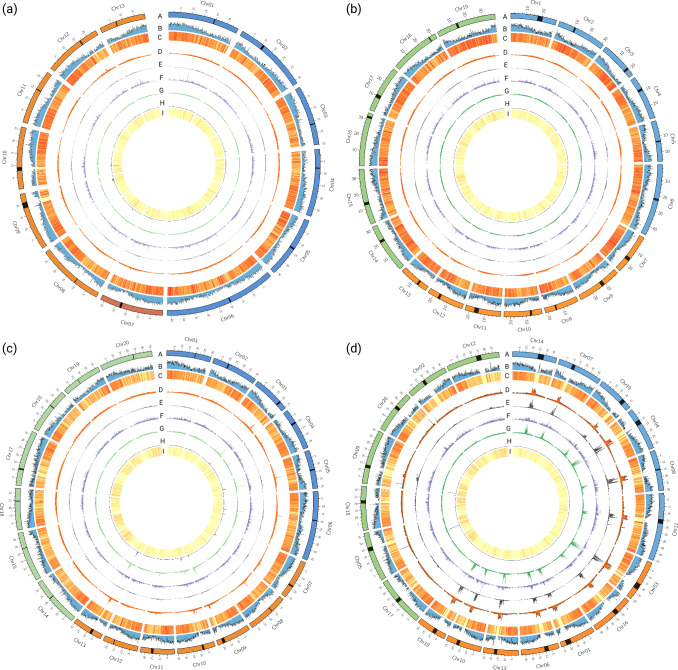
Genome of *Camelina* species. **a** *Camelina microcarpa* tetraploid Cmi4X, **b** *C. microcarpa* CmiT1, **c** *C. microcarpa* CmiT2, and **d** *C. sativa* CsaDH55. The outer track (A) represents chromosomes, with the subgenomes coloured in blue, orange, and green and the centromeres indicated by a black band. The remaining tracks from the outer to inner circle represent gene density (1 to 50 per 100 kb bin) (B), gene expression (log(FPKM + 1) (C), distribution per 100 kb bin of repeat elements (1 to 908) (D), gypsy elements (1 to 619) (E), copia elements (1 to 69) (F), Helitrons (1 to 600) (G), mutator elements (1 to 889) (H), and full-length LTR elements (1 to 7) (I). Note: For all *C. microcarpa* genomes the chromosomes are numbered from 1-13, 19 or 20; however, for *C. sativa* the chromosomes are numbered according to the original reference genome publication as in Kagale at al. (2014)), but are ordered according to their closest homologue in *C. microcarpa*.

The sequencing of the *C. microcarpa* and *C. sativa* genomes used both Pacific BioSciences (PacBio) and Oxford Nanopore Technology (ONT) long-read sequencing techniques. *Camelina microcarpa* CN119243 (Cmi4X) is a tetraploid, whereas the other three genotypes CN120025 (CmiT2), CN119205 (CmiT1) and DH55 (CsaDH55), are hexaploid in nature. Initial genome assembly was performed with 12-106× coverage of ONT, 14-36× PacBio HiFi, and 32× of PacBio CCS reads (Supplementary Table 1), which resulted in base assemblies with contig N50 lengths ranging from 3.4 to 19.82 Mb. Further, the base assemblies were scaffolded using chromosome conformation capture sequencing data (HiC) to generate contiguous assemblies with scaffold N50 lengths of 26.09 Mb, 29.88 Mb, 28.42 Mb, and 32.12 Mb for Cmi4X, CmiT2, CmiT1, and CsaDH55, respectively (Supplementary Fig. 1). The genomes comprised either 13, 19, or 20 pseudomolecules, respectively as expected based on the known chromosome numbers. The chromosomes were assigned to subgenomes (SG) and numbered according to the sequence similarity shown after alignment with the *C. neglecta* and *C. sativa* genomes as described in Chaudhary et al.^10^; such that SG1 (chromosome 1−6) is similar to both *C. neglecta* and the first subgenome of *C. sativa*, SG2 (chromosome 7−13) is similar to the second subgenome of *C. sativa*, and SG3 (chromosome 14−19/20) is the remainder of the genome (Fig. 1). The quality of the genomes were confirmed by various methods, including BUSCO analysis^24^ which identified between 99.2% and 99.5% of the Embryophyta universal single-copy orthologues in each of the genomes (Table 1). Further, Qualimap v.2.2.2^25^ analysis indicated more than 99.42% of available Illumina reads were mapped to the genome with a general error rate of 0.0027% (Supplementary Table 2). The assembled genomes comprised more than 91.04% of the estimated genome size using kmer analysis^26^ (Supplementary Fig. 2). The assembled genomes contain more than 93.76% of the genomic sequences in pseudomolecules or chromosomes for Cmi4X, 99.34% for CmiT2, 96.63% for CmiT1, and 96.38% for DH55. The representation of full-length long terminal repeat (LTR) elements was high for each of the genomes as measured by the LTR assembly index (LAI)^27^, ranging between 18.27 and 22.49, suggesting high-quality assembly of the repetitive fraction of the genome.

**Table 1:** Features of *Camelina microcarpa* and *C. sativa* genomes

Genomes	CN119243	CN120025	CN119205	DH55
Estimated genome size (Mb)	419.6	577.3	649.2	711.9
Assembled genome size (Mb)	384.06	555.63	607.97	678.31
No. of chromosomes (n)	13	19	20	20
Scaffold N50 (Mb)	26.09 (7)	29.88 (8)	28.42 (9)	32.12 (10)
Scaffold N90 (Mb)	19.45 (13)	22.87 (17)	23.24(19)	26.37 (19)
*N* counts	3500	258,100	11,200	3200
Number of repeat elements	230,176	322,719	403,779	616,027
Percentage of repeat elements	41.36	42.40	44.87	50.25
Total number of full-length LTR	1905	3194	4057	4576
Copia (%)	4.97	5.09	5.15	7.08
Gypsy (%)	11.80	12.93	13.03	12.39
Helitron (%)	15.60	15.90	17.28	19.95
Other repeats (%)	8.99	8.48	9.42	10.83
LAI	22.2	22.23	22.49	18.27
Total number of genes	65,354	95,759	101,172	102,393
BUSCO (%)	99.2	99.4	99.4	99.5

Repeat elements comprised 41.36%, 42.40%, 44.87%, and 50.25% of the assembled genomes of Cmi4X, CmiT2, CmiT1, and CsaDH55, respectively, where Gypsy (11.80–13.03%), Copia (4.97–7.08%) and Helitrons (15.6–19.95%) are the dominant classes of repeat elements (Supplementary Data 1). The number of full-length LTR elements as might be expected correlated with the genome size; utilizing these sequences to identify the superfamilies among the Copia and Gypsy elements, a similar pattern of prevalence was found in each of the *Camelina* genomes and sub-genomes, with the notable exception being the levels of Ale (Copia), Athila (Gypsy) and Tekay (Gypsy) elements in SG3 of the hexaploids (Supplementary Data 2). As a proportion of the total elements, the Ale elements decreased somewhat in the third sub-genome while the Tekay elements significantly proliferated (Supplementary Data 2, and Supplementary Fig. 3). The increase in Tekay elements in the third sub-genome is a recent expansion event (<2 mya) and would presumably have contributed to the evolution of the SG3 in each hexaploid. The pattern of repeat expansion is mirrored in the third subgenomes of *C. microcarpa* T1 and *C. sativa*, while the *C. microcarpa* T2 showed a lower percentage of Ale elements and an increase in Tekay elements, it also showed a unique small increase in Athila elements (Supplementary Fig. 3). The most obvious feature was the expansion of the total number of full-length LTR elements in the third sub-genome for all three hexploid species, although to a lesser extent in *C. microcarpa* T2, with the average prevalence increasing from one every ~200 Kb in any of SG1 or SG2, to one every ~100 Kb in SG3 (Supplementary Fig. 4). Based on the centromeric retrotransposon repeats (CRB) reported in *Brassica rapa*^28^, tentative centromeric positions were assigned with possible pseudo-centromeres identified in chromosomes of the third subgenome (Supplementary Table 3). It was noted that there was a proliferation of repeat elements around the centromeres of SG1 and SG2 for CsaDH55, particularly Gypsy elements and Helitrons. This was also observed, but to a lesser extent, for CmiT1, which shares the *n* = 20 karyotype, the same subgenome bias was not found for CmiT2 (Fig. 1, and Supplementary Data 2).

Helixer^29^, a machine learning-based de novo prediction tool for gene annotation, was used to predict gene and protein models in the respective genomes (Supplementary Data 3). We predicted 65,354 protein coding genes in Cmi4X, 95,759 genes in CmiT2, 101,172 in CmiT1 and 102,393 predicted genes in CsaDH55, with associated high confidence BUSCO scores (Supplementary Fig. 5). In the tetraploid and all three hexaploids, SG2 had the lowest gene number while SG3 had the highest, yet due to the subgenome size this equated to SG2 having the highest gene density while SG3 had the lowest (Supplementary Data 3). The tetraploid (Cmi4X) genome contained higher numbers of tandemly and proximally duplicated genes, from 2.4–3.8 fold and 1.6–2.3 fold greater, respectively, compared to the first two subgenomes in all hexaploids, which suggests either loss of such duplicates after the whole genome duplication process or the more recent proliferation of these genes in the tetraploid (Supplementary Data 4). There was a higher number of tandem and proximal duplicate copies in the third subgenome of the hexaploid *Camelina* species, compared to SG1 and SG2, ranging from a 1.35- to fourfold greater number (Supplementary Data 4). Gene fractionation was estimated using syntenic gene analysis; across all genomes 22,843 *Arabidopsis thaliana* syntenic orthologs were present, of these 93.40% were found in diploid *C. neglecta*^22^, while a reduction was seen to ~90% in all *C. microcarpa* subgenomes, the third subgenome of *C. sativa* (CsaDH55) appeared to show some level of fractionation with only 86.75% syntenic genes being present, although this was confounded by the increased presence of tandemly repeated genes, which were not necessarily identified by the synteny analyses (Supplementary Table 4).

Annotation of resistance genes can often be compromised by their mis-annotation as repetitive sequences^30^, thus the Resistance Gene Analogs (RGA) were analyzed for all the assembled genomes using RGAugury^31^ (Supplementary Data 5). The *C. microcarpa* T1 genome had the highest number of RGA compared to other genomes; however, both hexaploid *C. microcarpa* species possessed higher numbers of RGA clusters, containing at least 10 RGA (Supplementary Table 5). Receptor-like kinases (RLK) were the dominant class for all the species. Although there was some loss and gain of RGA in clusters across species, in general the clusters were found in conserved positions across the *Camelina* species. Closer inspection of the largest cluster, observed on chromosome 6 of *C. neglecta*, showed RGA expansion in the *C. microcarpa* species, and presumably a subsequent loss of genes in the derived *C. sativa* genome (Supplementary Fig. 6). The variable pattern of gene retention was invariably due to loss and gain of RLK loci.

### Evolutionary relationship among *Camelina* species

**Fig. 2:**
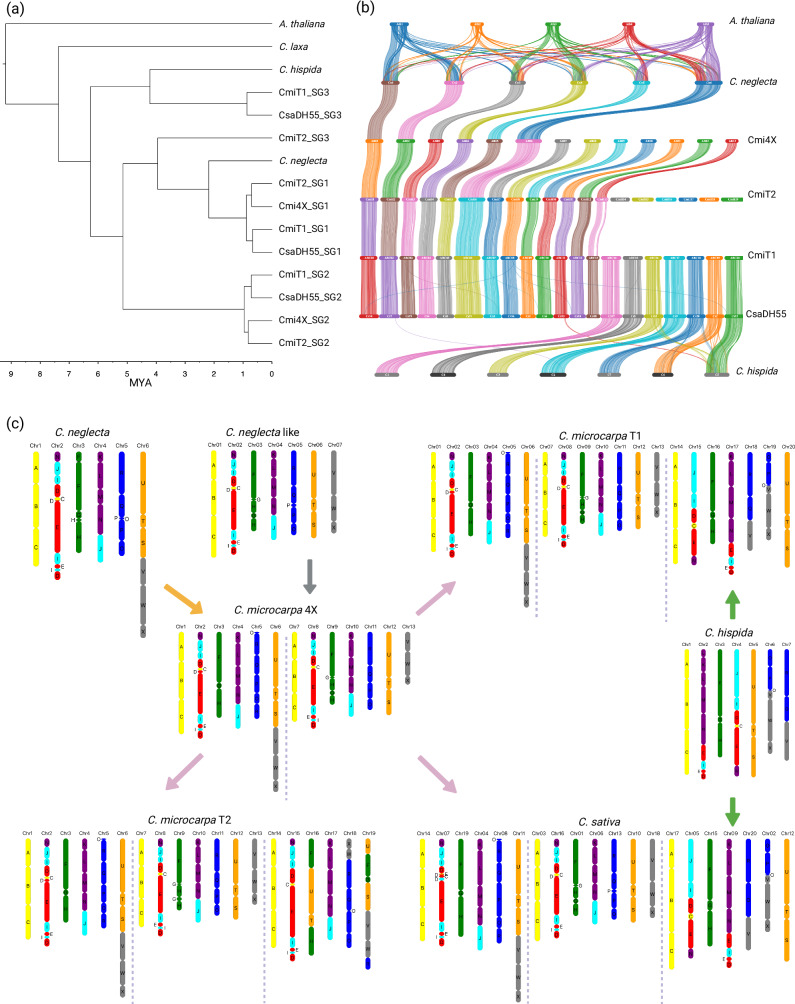
The evolution of *Camelina* species. **a** The species tree represents the relationship among different subgenomes and species of *Camelina* with approximate age of divergence in million years ago (MYA); **b** A syntenic relationship among different *Camelina* species and *Arabidopsis thaliana* showing relationship between subgenomes across *Camelina* species; and **c** Genomic block arrangements in *Camelina* species and probable evolutionary trajectory across various ploidy levels of *Camelina* species. CmiT1 is *C. microcarpa* Type 1, CmiT2 is *C. microcarpa* T2, Cmi4X is tetraploid *C. microcarpa*, and CsaDH55 is *C. sativa*.

The evolutionary relationships among the *Camelina* species were studied with different approaches, where the four genomes from this study, along with *C. neglecta*^22^, *C. hispida*^23^, and *C. laxa*^23^ were utilized. First, assembled genomes were compared to observe the collinearity among different *Camelina* species using whole genome alignment (Supplementary Fig. 7). The relationship between the lower chromosome number species and the hexaploids was as expected based on previous genotypic and cytogenetic work^10^, yet the chromosome structure was incredibly conserved with very limited large structural variation differentiating the genomes. Cluster analysis using Orthofinder v.2.5.2^32^ further confirmed the relationships among the species (Fig. 2a), with SG1 of *C. sativa* (CsaDH55), *C. microcarpa* (Cmi4X, CmiT1, CmiT2) and *C. neglecta* clustering together; similarly, SG2 of the tetraploid and the three hexaploids were clustered. Interestingly, while SG3 of *C. sativa* (DH55) and *C. microcarpa* T1 (CN119205) showed a close relationship with *C. hispida*, the third subgenome of *C. microcarpa* T2 formed a separate clade relatively close to the *C. neglecta* genome. While the outgroups of *A. thaliana*, *C. laxa*, and *C. hispida* possessed higher numbers of unique orthogroups, SG3 from the *C. microcarpa* T2 hexaploid ranked just below them, and *C. sativa* DH55 possessed the lowest number of species-specific orthogroups for all the subgenomes (Supplementary Data 6).

Syntenic analysis was performed using MCScanX^33^ based on ancestral genomic blocks reported in *A. thaliana*^34^; all 24 genomic block (GBs) were identified in all genomes (Supplementary Data 7). The visualized syntenic analysis^35^ corroborated the conserved nature of the genome structure among the species, including all rearrangements in SG1 and SG2 relative to the ancestral genome (Fig. 2b, and Supplementary Data 7). The GB arrangement for SG1 of all the assembled higher ploidy genomes is similar to *C. neglecta*, except for chromosome 5, which shows evidence of rearrangement (Fig. 2c). *C. hispida* showed strong synteny with SG3 of *C. microcarpa* T1 (CmiT1) and *C. sativa* (CsaDH55), which would align with the suggestion that *C. hispida* could be an extant progenitor for SG3 of *n* = 20 *Camelina* species; however, there has been a conserved rearrangement of GBs of chromosome 2 of *C. hispida* relative to the hexaploids (Fig. 2c). The origin of the lower chromosome number SG3 of *C. microcarpa* T2 has yet to be found based on the now characterized genomes of the *Camelina* genus; although this subgenome shows strong similarity with *C. neglecta* and hence SG1 of the other *Camelina* species, significant reshuffling of ancestral GBs has occurred for chromosomes 16, 18 and 19 (Fig. 2c). Multiple recombination events would need to be inferred between chromosome 3, 5 and 6 of a *C. neglecta* like genome to result in the final chromosomes 16, 18 and 19 of *C. microcarpa* T2 (Supplementary Fig. 8).

**Fig. 3:**
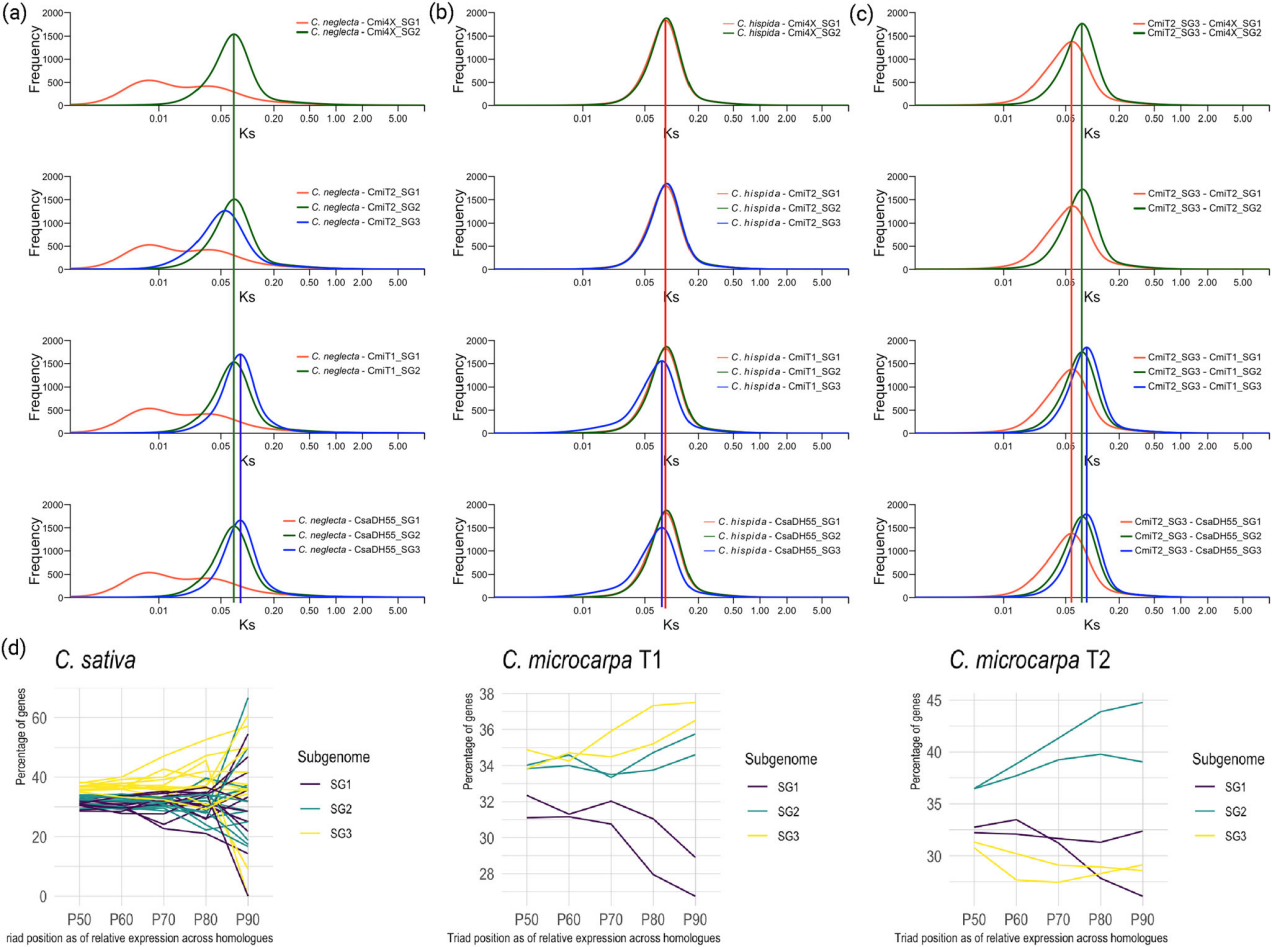
Subgenome evolution in *Camelina* species. Ks distribution of *Camelina neglecta* (**a**), *C. hispida* (**b**), and *C. microcarpa* T2 (**c**) relative to subgenomes of other *Camelina* species, the legend shows the genepairs of the subgenomes from which Ks analysis were performed; and **d** Subgenome dominance analysis in *Camelina* species, the plots shows percentage of genes for particular subgenome (SG1: Purple, SG2: Blue, and SG3: Yellow) with a biased gene expression of at least 50–90% (P60: 60%; P70:70%; P80:80% and P90:90%) in a triad comparison among the subgenome orthologues. *Camelina sativa* shows expression for 12 tissues from *C. sativa* var. DH55 (adopted from Kagale et al.^14^), *C. microcarpa* T1 shows leaf tissue data for two genotypes (TMP26168 and CN 119205) and *C. microcarpa* T2 show leaf tissue data for two genotypes (CN120025 and CN115248). In all instances, three replicates were sequenced. Source data are provided as a Source Data file.

The syntenic gene pairs identified among the subgenomes of the different *Camelina* species were utilized to analyze the synonymous substitution rate (Ks) using the GenoDup pipeline^36^. As previously reported in Chaudhary et al.^22^, SG1 of all higher ploidy *Camelina* species diverged from *C. neglecta* at approximately the same time, similarly SG2 of all higher ploidy *Camelina* species diverged from *C. neglecta* around the same time (Fig. 3a). In the same comparisons with *C. neglecta*, SG3 of CmiT1 and CsaDH55 showed a common older peak compared to that of SG2, which was in contrast to SG3 of CmiT2 where the peak suggested a more recent divergence from *C. neglecta* (Fig. 3a). On comparison with *C. hispida*, the other potential progenitor diploid genome, there was no clear differentiation between the subgenomes of Cmi4X, as well as the first two subgenomes of each of the hexaploids. However, for CmiT2 all three subgenomes shared a peak of divergence, which again differentiated this species from both CmiT1 and CsaDH55, where SG3 showed a more recent divergence event from the progenitor (Fig. 3b). Comparing Ks rates among higher ploidy *Camelina* species, identified a barely indistinguishable peak for the first and second subgenomes of all three hexaploids, either when compared with the tetraploid or when compared among the hexaploids (Supplementary Fig. 9). However, SG3 of CsaDH55 showed two equal peaks when compared with SG3 of CmiT1, but not CmiT2. Although *C. sativa* and CmiT1 appear to share a common progenitor for SG3, the additional peak could suggest that the *C. hispida-*like species was added to a tetraploid genome in two different events, leading to separate lineages for *C. sativa* and CmiT1 (Fig. 3c).

### Influence of chromosomal architecture on gene expression dominance

Subgenome dominance analysis or variance of gene expression (FPKM) was performed with the sets of triplicated orthologous genes (17,378−19,278 triplicates) identified among hexaploid *Camelina*, with two or three genotypes and different tissues of each species used. The data from *C. sativa* (DH55 and CN120013) were mapped to the CsaDH55 reference genome, while that of *C. microcarpa* T1 (CN119205 and TMP26168) was mapped to the CmiT1 genome reference, and that of *n* = 19 chromosome *C. microcarpa* T2 data (CN120025 and CN115248) was mapped to the CmiT2 reference. The third or second subgenome was found to show higher levels of gene expression dominance for all genotypes with *C. sativa* type genome structure, whereas, for *C. microcarpa* T2 (*n* = 19), the second subgenome was consistently found to show dominant gene expression over the other two subgenomes (Fig. 3d, Supplementary Figs. 10 and 11, and Supplementary Data 8). These results might indicate that subgenome gene expression dominance in *Camelina* species was determined by the nature of the progenitor genome hybridized with the tetraploid structure, rather than the order of events.

**Fig. 4:**
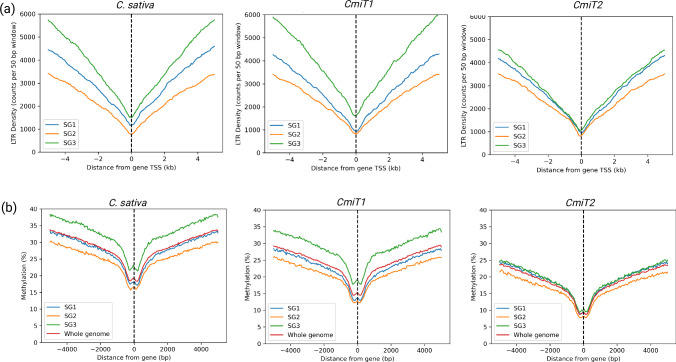
Distribution of LTR retrotransposons and CpG DNA methylation around annotated genes. **a** LTR retrotransposon density around gene transcription start sites (TSS) in *Camelina sativa*, *C. microcarpa* T1 (CmiT1), and *C. microcarpa* T2 (CmiT2) genomes. The *x*-axis shows the distance from the gene TSS (±5 kb), and the *y*-axis represents LTR density measured as counts per 50 bp window. The three subgenomes are shown as SG1 (blue), SG2 (orange), and SG3 (green). **b** DNA methylation levels around genes in *C. sativa*, CmiT1, and CmiT2. The *x*-axis shows distance from genes (±5 kb), and the *y*-axis indicates CpG methylation percentage. Lines represent SG1 (blue), SG2 (orange), SG3 (green), and the whole-genome average methylation rate (red).

To further understand the nature of the subgenome architecture, different genomic factors were assessed across the three subgenomes in each hexaploid species. The analyses of repetitive sequences across the three species and their respective subgenomes had identified some variation, in particular with regard to the type of elements that had proliferated in the different species. All species showed a higher density of LTR elements in SG3, although this was more pronounced in *C. sativa* and CmiT1 (Supplementary Data 2). The distribution of such elements proximal to the annotated genes was measured for each species and subgenome (Fig. 4a). The three species demonstrated similar patterns of LTR density, with SG3 showing the highest and SG2 the lowest levels. ONT and PacBio HiFi data for each species were utilized to call CpG methylation status across the three hexaploid genomes; the overall methylation status across the genome was 31% to 46%, with *C. sativa* showing the highest level. The pattern across the three subgenomes in each species was the same, with the methylation status from lowest to highest being SG2 < SG1 < SG3. (Supplementary Data 9). In order to assess potential impact on gene expression, the CpG methylation status was assessed across the gene body, and 5Kb up and downstream of each annotated gene in the three subgenomes for each species (Fig. 4b). The expected pattern of increasing methylation with distance from the gene start and end coordinates was observed in all instances. The patterns among the subgenomes were similar among the species, with SG3 showing the highest and SG2 showing the lowest methylation density in each species. There were subtle differences, with CmiT2 showing less differentiation between the subgenomes. Overall, the methylation densities mimicked the LTR densities as might be expected, since most LTR elements were methylated at >80% of their CpG sites (Supplementary Data 10).

**Fig. 5:**
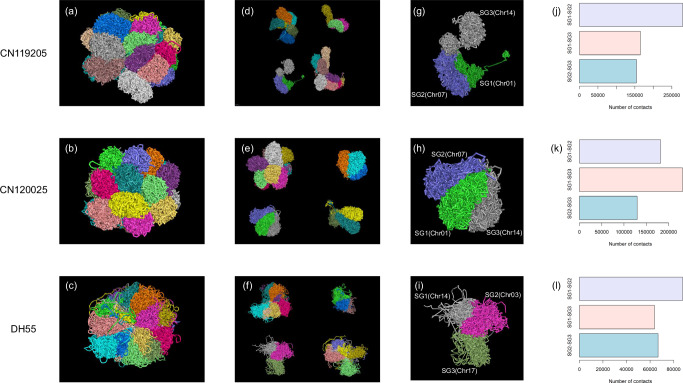
3D structure of *Camelina* species showing interaction among chromatids as well as homoeologous chromosomes. **a**–**c** 3D structure of the whole genome; **d**–**f** Drag-out of homologous chromosomes; **g**–**i** Magnification of three homologous chromosomes; **j**–**l** Comparison of the number of contacts between different homologous chromosomes. CN119205 is CmiT1, CN120025 is CmiT2 and DH55 is CsaDH55 genome. All figures utilised Omni-C data. Source data are provided as a Source Data file.

The HiC data generated for the assembled genomes, from which physical chromosomal proximity can be inferred, were utilized to assess potential relationships between the subgenomes of the polyploid species. The Iterative Correction of HiC data (ICE) normalized matrices were used to identify the number of significant interactions within and between the chromosomes using fithic2^37^. A total of 684,083 significant interactions (*q*-value < 0.01) were found in the tetraploid Cmi4X, where intra-chromosomal interactions dominated at 90.1% while inter-chromosomal interactions were 9.9%, of which 42.8% were between sub-genomes, and there were less significant interactions among the chromosomes of the second sub-genome compared to the first (Supplementary Fig. 12a, Supplementary Table 6 and Supplementary Data 11). In CmiT2, 88.93% and 11.06% of the 2,090,111 significant interactions were intra- and inter-chromosomal, respectively (Supplementary Fig. 12b, and Supplementary Data 12). SG1 and SG3 showed the highest number of inter-chromosomal interactions (67.97%) among the sub-genome pairs. In the case of CmiT1, a total of 998,360 significant interactions were found, among these 89.04% are intra-chromosomal and 10.9% are inter-chromosomal (Supplementary Fig. 12c, Supplementary Table 6, and Supplementary Data 13). Among the sub-genome pairs SG1 and SG2 showed the highest number of inter-chromosomal interactions, 37.09% compared to 31.53% between SG1 and SG3 and 31.38% between SG2 and SG3. Interestingly, after filtering of a similar amount of HiC data, a smaller number of significant interactions could be detected for CsaDH55; a total of 147,541 interactions were found and among these, 90.86% were intra-chromosomal while 9.14% were inter-chromosomal (Supplementary Fig. 12d, Supplementary Table 6, and Supplementary Data 14). Almost all the inter-chromosomal interactions were between homoeologous chromosomes, and similar to CmiT1, SG1 and SG2 showed the highest number of significant interactions (36.3%), while SG3 interacted less with both SG1 (33.5%) and SG2 (30.2%). It was evident that homoeologous chromosomes have some level of association and presumably proximity in the nucleus of the polyploids. Three-dimensional images which visualize the chromosome positions within the nucleus, effectively simulating their proximity and the interactions among the homoeologous chromosomes in *Camelina* species, were generated from chromatic capture information using NucProcess^38^ (Fig. 5). As might be expected, the chromosomes each formed clear domains, and the differential interactions between the sub-genomes could be visualized. In order to confirm the observed differences, further filtering of the significant interactions derived from fitHiC was carried out, removing those bins with interactions >1000, which could represent erroneous read mapping to repetitive regions. In addition, additional HiC data generated using an alternative method (Proximo compared to Omni-C) was analyzed using the NucProcess method (Supplementary Table 6). In all instances, CmiT1 and CsaDH55 showed higher levels of interactions between SG1 and SG2, while CmiT2 showed higher levels of interactions between SG1 and SG3.

### Potential to exploit the genetic diversity of *Camelina microcarpa* Type 2

**Fig. 6:**
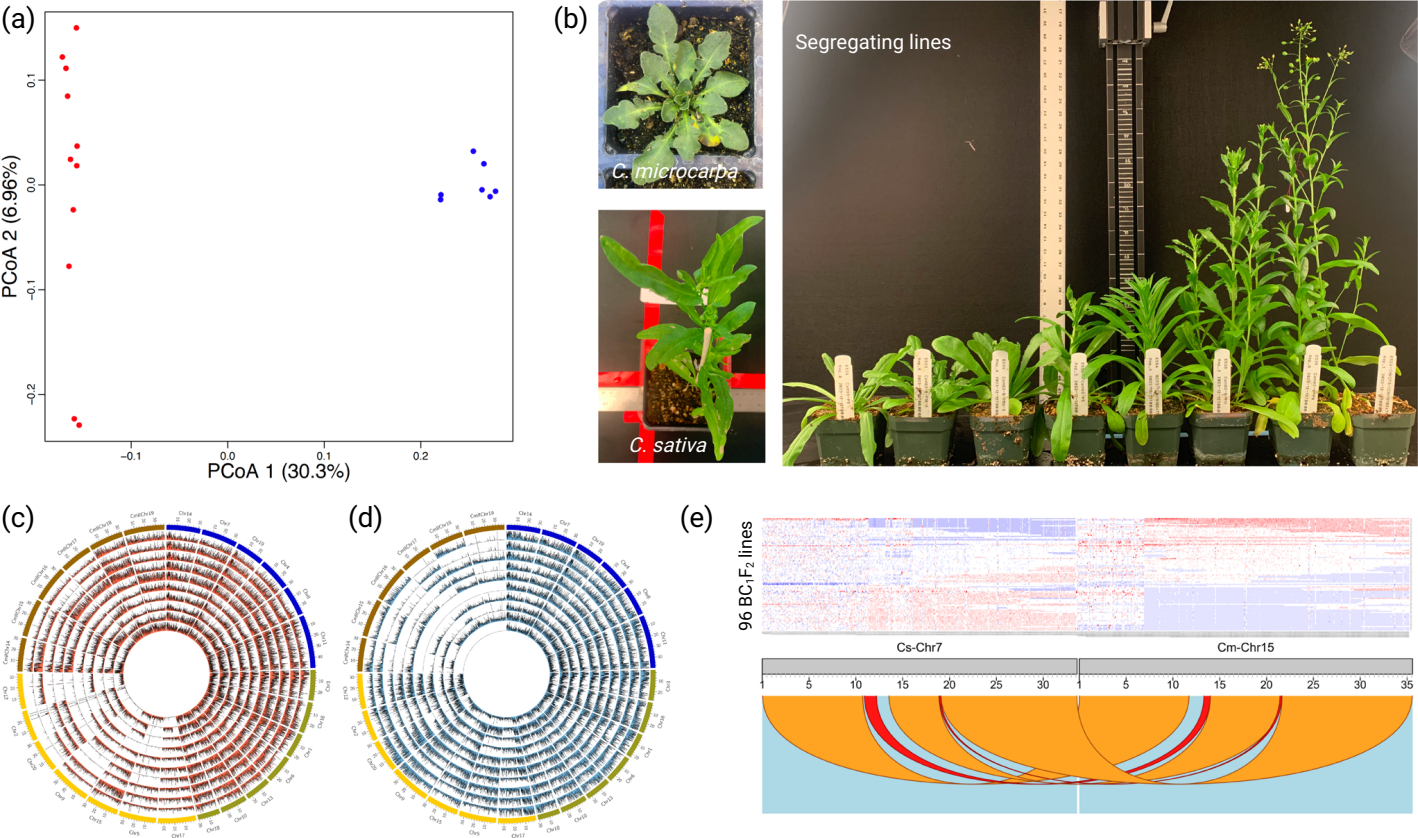
Utilization of *Camelina microcarpa* T2 to increase genetic diversity. **a** Principal Coordinate Analysis among the CmiT2 lines to represent genetic diversity; **b** Leaf morphology of segregating plants developed from interspecific hybridization between *Camelina microcarpa *× *Camelina sativa*; Distribution of reads mapped to the pseudo-genome coming from segregating lines F_2_−83-8 (**c**), F_2_-82-6 (**d**), the blue, green and yellow bands in the outer track represent SG1, SG2 and SG3 from CsaDH55, and the brown bands represent SG3 of CmiT2, inner individual tracks represents reads from different F_2_ lines mapped to the pseudo-genome; and **e** homoeologous recombination in 96 segregating lines as represented by loss/gain of reads represented by blue/red colour on top, individual rows represent different segregating lines, the syntenic relationship between homoeologous chromosomes are represented by links at the bottom of the image.

Genetic diversity among 18 *C. microcarpa* T2 genotypes was analyzed against the CmiT2 reference using genotype by sequencing (GBS) data from Chaudhary et al.^10^. The species previously had been noted to have higher genetic diversity compared to *C. sativa* and the principal coordinate analysis showed a higher dissimilarity among these genotypes, with 30.3% of variation on PC1 and 6.96% of variation on PC2, supporting two subpopulations (Fig. 6a, and Supplementary Fig. 13). The differentiation was also high (*F*_ST_ = 0.221) between these two subpopulations (Supplementary Table 7).

In order to assess the feasibility of capturing some of the available diversity from the wild relative, hybridization of CmiT2 with *C. sativa* was performed. A total of 49 F_2_ plants were obtained from four F_1_ progenies from a cross between *C. microcarpa* T2 (CN119102) and *C. sativa* (CN120019), of which four plants showed spring-type early seedling growth, while the remainder of the plants were winter-type. There was a marked difference in leaf shape, leaf waxiness and number of flowers in the segregating plants in comparison to the parents (Fig. 6b). Among the 49 plants, only 7 were completely fertile, 3 were partially sterile and the remainder were sterile (Supplementary Data 15). It was anticipated that homologous chromosome pairing and recombination would occur between the common subgenomes 1 and 2, but the disparity between the third subgenomes, which differentiates the parental lines, would result in some abnormal chromosome pairing. Genetic analysis of the F_2_ lines using GBS data, where sequence reads were mapped to a pseudo-genome containing all four sub-genomes (see “Methods”), indicated that SG1 and SG2 did pair relatively normally, but a non-random mapping of sequence reads was observed for SG3. The segregating plants showed a bias towards retention of either SG3 from *C. sativa* or CmiT2, according to the parental hybrid from which the progeny were derived (Fig. 6c, d), this bias was also reflected in the leaf morphology of the progeny (Supplementary Fig. 14). A number of the plants showed missing chromosomal segments either due to deletion or translocation, probably resulting from homoeologous recombination.

In order to further assess recombination events between *C. sativa* (CN120019) and *C. microcarpa* (CN119102), one of the hybrids was backcrossed with PI650132 (a spring-type *Camelina alyssum*, considered a cryptic species of *C. sativa*). The resulting backcross population was self-pollinated to generate BC_1_F_2_ seeds; of these, 96 lines were genotyped using GBS (Fig. 6e). Mapping of sequence reads from the 96 individuals to the pseudo-genome identified a similar overall pattern with biased retention of the third subgenome of the *C. sativa* type. Studying some of the homoeologous regions, there was evidence of exchange; a uniform distribution of mapped reads was observed across chromosome 15 of CmiT2, yet based on read depth, the reads segregated for the absence (AA), single copy presence (AB) and presence (BB), of homoeologous regions of chromosome 15 and chromosome 7 of CsaDH55, respectively, suggesting a homoeologous recombination event between these chromosomes (Fig. 6f). Beside this event, some of the lines were found to be missing whole chromosomes such as chromosome 17 and chromosome 2 of *C. sativa*.

## Discussion

Genome assemblies and associated resources are important tools in the study of polyploids, and identifying relationships among closely related species can reveal distinguishing genomic features, which shape their genome evolution. Previously, genome assemblies of two diploid *Camelina* species had shed light on the evolutionary history of *C. sativa; C. neglecta* was identified as a progenitor of the first subgenome of hexaploid *C. sativa*, and it had been suggested that *C. hispida* was an extant progenitor of the third subgenome^10,39^. In the current study, three high-quality genome assemblies of *C. microcarpa*, of which one is a tetraploid, and the other two are hexaploids differentiated by their chromosome number, have provided further insights into the evolution of the *Camelina* genus. The tetraploid *C. microcarpa* genome, which coincides with the first and second subgenomes of *C. sativa* and both *C. microcarpa* hexaploids, suggested a step-wise merger of species from diploid (*n* = 6) to tetraploid (*n* = 13) and then hexaploid (*n* = 19 and *n* = 20)^22,40^. Yet the third subgenome of *C. microcarpa* T2, not withstanding the reduction in chromosome number from the common *n* = 7 to *n* = 6, is quite distinct in structure. Although the third subgenome of *C. microcarpa* T2 shared the same number of chromosomes with *C. neglecta*, a number of significant chromosomal rearrangements distinguish the genomes (Fig. 2). The Ks analyses suggests a more recent divergence from a *C. neglecta-*like genome compared to SG3 of the other *Camelina* hexaploids, and as yet no extant relative has been collected that resembles this subgenome structure.

The third subgenome of all hexaploids displayed a number of differences, which might relate to their later addition in the evolutionary trajectory of *Camelina* species. There was a higher gene number and the notable proliferation of full-length LTR in the third subgenome; the age of divergence of tandem duplicate genes indicated the third subgenome was added last to the tetraploid like species on the path to the hexaploids (Supplementary Fig. 15). Although the rate of synonymous substitutions indicated that the third subgenome from *C. microcarpa* T2 was more recently diverged from *C. neglecta* compared to the second subgenome, the pattern of increased tandem duplicates in the third subgenome also reflected that seen in *C. microcarpa* T1 and CsaDH55. It was proposed that *C. sativa* evolved from a *C. microcarpa* T1-like genome^9,39^; this could not be reconciled with the observation that the third subgenome of *C. sativa* and the third subgenome of *C. microcarpa* T1 showed multiple peaks of divergence. These data suggest two separate lineages, where a *C. hispida*-like genome hybridised at different time intervals with the tetraploid structure to form the *n* = 20 chromosome *Camelina* species. A chloroplast-based phylogenetic tree derived from the genome data also suggested different potential progenitors for the formation of *C. sativa* and *C. microcarpa* T1 (Supplementary Fig. 16). Thus, we speculate that although *C. sativa* and *C. microcarpa* T1 share subgenome structures, they represent independent evolutionary events.

It is interesting to note that to be in agreement with the two-step hypothesis for the formation of a hexaploid, similar to that inferred in the evolution of related Brassica paleohexaploids^11^, the last genome added, SG3, should show dominant gene expression. In the case of *C. sativa* and *C. microcarpa* T1, which shared the same subgenome structure, this was true in most instances, with the third subgenome often showing the expected expression dominance. However, interestingly, in some tissues SG2 showed gene expression dominance; this variance could be a reflection of the age of the evolutionary events that led to the species formation, with such young genomes only beginning to develop patterns of genome differentiation. In the case of *C. microcarpa* T2, the second subgenome was consistently found to show gene expression dominance. The mechanism of gene expression dominance has been studied in a number of species and suggested to be associated with a number of genome features within the diploid progenitors, including genome compaction, distribution of repetitive elements and chromosome architecture^41^. In *Camelina*, there was no such obvious correlation, since each of the hexaploid species was similar in basic organization, with the third subgenome for all showing a higher gene number, a higher number of recently-proliferated full-length LTR elements, and a corresponding higher level of CpG methylation, while SG2 showed the opposite. The most notable association with the observed gene expression dominance was that of biased inter-chromosomal interactions for the subgenomes in the hexaploids. Chromosomal 3D architecture has been consistently shown to impact gene expression^42^, and the dominant genome, either SG2 in *C. microcarpa* T2 and SG3 for *C. microcarpa* T1 and *C. sativa*, had the least number of significant interactions with their fellow subgenomes, suggesting proximity in the nucleus could be dictating gene expression. It has been shown in a number of species that proximity, as determined through chromosome capture, suggests co-localised homoeologous genes share common expression patterns, which is also reflected in other measures such as methylation status and histone modification patterns^43,44^. This would be in concurrence with the two non-dominant sub-genomes showing limited evidence of expression bias. Interestingly, the alignment of chromosomal proximity with genome dominance has been suggested from work in other allopolyploid species. In cotton, it was noted that homoeologous gene pairs with extreme expression bias were found to show fewer chromatin interactions^45^. In wheat, when comparing gene expression of syntenic triads from the A, B and D genomes across multiple tissues, there was shown to be a subtle but consistent bias to the D genome^46^ and in Concia et al.^47^. It was found that the A and B genomes interact more frequently than any of the other possible pairings. Thus, it appears that genome-wide expression dominance could result from the higher-order organization of the chromatin in the nucleus. This could be driven by the level of homology between the underlying subgenome chromosomes; for CmiT2, the high level of homology observed between SG1 and SG3 may influence their proximity in the nucleus and, by exclusion, allow SG2 to have greater regions of accessible chromatin.

The identification of the unique species of hexaploid *C. microcarpa* Type 2, further the prospects of increasing genetic diversity in the *C. sativa*, which has a very low genetic base^3,4,10^. Although the clear differentiation of genome structure does raise the issue of likely chromosomal imbalance and thus reduced fertility when trying to cross the two species, which was almost certainly the reason for the anomalies observed in previous studies, which attempted interspecific hybridization prior to the identification of two *C. microcarpa* species^48,49^. Yet despite this, as shown here, homoeologous recombination with *C. sativa* can result in the capture of genetic diversity from *C. microcarpa* T2. The much higher levels of genetic variation and potential phenotypic variation for traits of interest in *C. microcarpa* T2 suggest further efforts to encourage homoeologous recombination and stabilise the progeny are worth investigation. As knowledge of factors limiting homoeologous recombination in other related species is uncovered^50,51^, new strategies to affect this work may become feasible.

## Methods

### Plant material for DNA extraction and sequencing and assembly

For *C. microcarpa* (accession: CN120025), fresh leaf tissue was flash frozen in liquid nitrogen and stored at −80 °C before DNA extraction. Leaf material was sent to Dovetail Genomics (Scotts Valley, CA, USA); high molecular weight (HMW) DNA extraction and long read sequencing using PacBio, followed by HiC scaffolding, were performed by Dovetail Genomics^22^. For the accessions CN119243, CN119205, and DH55 nuclei were extracted as described in Workman et al. (10.17504/protocols.io.4vbgw2n). Briefly, fresh leaf tissue was homogenized in 200 mL of ice cold 1× HBS containing 0.15% β-mercaptoethanol and Triton X-100 using a kitchen blender. The homogenate was filtered through layers of cheesecloth and one layer of Miracloth, followed by centrifugation at 2500 × *g* at 4 °C for 20 min. DNA purification from isolated nuclei was carried out using the Nanobind Plant Nuclei Big DNA kit according to the manufacturer’s instructions (Circulomics, Menlo Park, CA, USA). DNA was size selected for >5 kb on a BluePippinTM (Sage Science Inc., Beverly, MA, USA) and Oxford Nanopore Technology (ONT) libraries were prepared using Ligation Sequencing Kit V14 (SQK-LSK114) and sequenced with R10.4.1 MinION flow cells on a GridION or MinION platform from ONT. HMW DNA for the same samples was also extracted using NucleoBond® HMW DNA kit (Macherey-Nagel) and sequenced on a PacBio Sequel IIe sequencing platform at the Global Institute for Food Security, Saskatoon, Canada (Supplementary Table 1). Illumina short reads, paired-end (PE) 2 × 125 bp, for three accessions (CN119243, CN120025, and CN119205) were generated using the HiSeq2000 Illumina platform (Illumina, San Diego, CA, USA). HiC libraries were prepared using the Dovetail® Omni-C® kit (Cantata Bio, Scotts Valley, California, USA) and the Proximo^TM^ Hi-C kit (Phase Genomics, Seattle, WA, USA) according to the manufacturer's protocol; the libraries were sequenced on the Illumina NovaSeq6000 platform (PE 2 × 150 bp).

Paired Illumina reads were used to estimate the genome size based on k-mer analysis using Jellyfish^26^ followed by visualization using GenomeScope^52^. The reads were filtered against the *C. sativa* chloroplast genome (Accession: NC_029337)^53^ using Bowtie2^54^ before processing for genome size estimation. In the case of DH55, paired raw reads (SRR1171872 and SRR1171873) from Kagale et al.^13^ were used. The hexaploid CN120025 was assembled using ~4 million CCS reads (~32.9× coverage clean reads) and the Falcon assembler, followed by HiC scaffolding using the Chicago HiRise platform (Dovetail Genomics, Scotts Valley, CA)^22^. For the tetraploid (CN119243) and hexaploids (CN119205 and DH55), HiFi data along with ONT data (parameter: --*ul*) were used to assemble the genome using Hifiasm v0.19.5 with default parameters^55^ (Table 1). HiC data generated using omni-C protocol were mapped to the base assemblies using HiC-Pro^56^ and scaffolding was performed using the EndHiC pipeline^57^. The hic contact maps generated by 3D-DNA^58^ were visualized in Juicebox^59^ and manual correction of misassembled regions was performed. Using the fitHiC tool (https://github.com/ay-lab/fithic), inter- and intra-chromosomal interactions were calculated as described by Zhang et al.^44^, with minor modification. Briefly, contact matrices for both intra- and inter-chromosomal interactions at 100-kb resolution were processed using fitHiC. The corresponding cumulative probability (*P*-value) and false discovery rate (*q*-value) were calculated for each interaction. Interactions with both *P*-value and *q*-value less than 0.01 and a contact count greater than 2 were considered significant.

### RNA extraction, sequencing, and sequence analysis

RNA extraction was performed for two CmiT2 genotypes (CN120025 and CN115248), two *C. sativa* genotypes (DH55, CN120013), two CmiT1 genotypes (CN119205 and TMP26168) and CN119243 (*n* = 13 chromosomes) with three replications for each. Seeds were grown in CYG seed germination pouches (Mega International, Newport, MN, USA) at room temperature for 1 week before total RNA extraction from leaf tissue for all samples except CN119243, TMP26168, and CN115248, which were sampled from the greenhouse. Total RNA was extracted using a standard RNeasy Plant Mini Kit (Qiagen) as described by the manufacturer with on-column DNA digestion. RNA-seq libraries were constructed using the TruSeq RNA preparation kit (Illumina) with 100 ng of RNA for each sample followed by sequencing on an Illumina HiSeq2000 platform (2 × 125 bp). Raw reads were filtered for low quality, short reads and adapter contamination using Trimmomatic v.0.33^60^. All trimmed reads were aligned with the respective reference genome using STAR v.2.7.6a^61^ using default parameters, except for *--alignIntronMax* set at 10000 and *--outFilterMismatchNmax* set at 4.

### Annotation of repetitive elements, gene prediction and centromeric repeat prediction

Helixer^29^ was used to annotate all the genomes using the model *land_plant* and without repeat masking. Repeat elements were annotated using an Extensive de-novo TE annotator pipeline v.2.1.0^62^, which uses LTRharvest^63^, LTR_finder^64^, LTR_retriever^65^, TIR_learner^66^, Generic repeat finder^67^ and HelitronScanner^68^. TEsorter^69^ was used to classify the LTR families based on the output from EDTA. A higher abundance of predicted centromeric repeats was used to position the centromeric regions^22^.

### Assembly quality assessment

Genome quality was assessed using BUSCO v.5.4.3^24^ utilizing embryophyta_odb10 (*n* = 1614) to compare the completeness of single-copy orthologs. Further, assembly quality was tested by aligning whole-genome Illumina short reads to the assemblies using BWA^70^, and the mapping quality was assessed using Qualimap v.2.2.2^25^. LTR assembly index (LAI)^27^ was calculated to assess the level of full-length LTR repeats as a further measure of genome quality.

### Methylation analyses

For ONT sequence data, basecalling and read-alignment were performed using Dorado (v1.3.0) with the parameters --model hac --modified-bases 5mCG_5hmCG to identify epigenetic modifications in the raw reads. The resulting alignment BAM file was sorted and processed with modkit (v.0.2.5) software using default parameters to generate summary counts of methylated and unmethylated cytosines in bedMethyl file format. In the case of HiFi data, PacBio HiFi reads were aligned to the respective genome using pbmm2 v.1.17.0 to produce a coordinated -sorted BAM file. CpG methylation was subsequently detected from the aligned HiFi reads using CpG-tools (aligned_bam_to_cpg_scores). Methylation was calculated across genomes and subgenomes as described in Parkin et al.^71^; in short, a minimum criteria of 10 valid reads and >=50% modified bases was used, with final methylated cytosines identified using the binomial probability distribution at a false discovery rate of <5%.

### Syntenic analysis

Syntenic analysis was performed to compare gene contiguity among species, using *A. thaliana*, *C. neglecta*, *C. microcarpa* T1, *C. microcarpa* T2, *C. sativa*, and *C. hispida*^23^. Protein models predicted from all three assembled genomes were reciprocally blasted against *A. thaliana* to define gene pairs and DAGChainer^72^ was used to identify syntenic blocks, which were assigned to a Cruciferae ancestral genomic block as defined by Schranz et al.^34^. Custom scripts were used to draw karyotype blocks for all the assembled genomes, and the syntenic genes were visualized using the online platform SynVisio^35^.

### RGA, orthogroups and duplicate gene analysis

The protein models for each genome were used to predict RGAs using the *RGAugury* pipeline v. 2.1.3^31^. RGA gene clusters were identified as described in Bayer et al.^30^. Protein models were used for orthogroup identification using Orthofinder v.2.5.2^32^, orthogroups were compared among *Camelina* species viz. *C. microcarpa* T1, *C. microcarpa* T2, *C. hispida*, *C. laxa*, *C. neglecta* and *C. sativa*, and duplicated genes were identified using DupGen_finder pipeline^73^, which identified tandem duplicates, proximal duplicates, transposed and whole genome duplicate gene pairs.

### Estimation of genomic age of divergence

*C. neglecta*^22^ and *C. hispida*^23^ were utilized to predict the age of divergence among different subgenomes of *Camelina* species. The rate of synonymous substitutions (Ks value) for each syntenic gene-pair among different species of *Camelina* was calculated using GenoDup pipeline^36^, which uses TranslatorX^74^ and MAFFT^75^ for multiple sequence alignment, and PAML^76^ for calculating the rate of synonymous substitution between gene-pairs. The Gaussian mixture model from R package mclust^77^ was used to plot the distribution of Ks and identify the number of Gaussian components. Based on these components, the mean value of Ks distribution was identified and used to calculate the age of divergence as described by Kagale et al.^13^. Further, MCMCtree^78^ was used to confirm the age of divergence from the single copy orthologs from subgenomes of *Camelina* species and *A. thaliana,* where the divergence of *A. thaliana* with *Camelina* species was assumed as 9.9 million years ago^79^.

### Subgenome gene expression dominance analysis

Subgenome gene expression dominance was analyzed for at least two genotypes of each of the hexaploids *C. sativa, C. microcarpa* T1 and *C. microcarpa* T2. Additional RNA-Seq data for *C. sativa* DH55 derived from 12 tissue types was adopted from Kagale et al.^14^. Further additional RNA-Seq data was generated as described above for three replicated sets of etiolated seedlings for the three hexaploids, where 10 seeds were germinated and the hypocotyl extended in the dark for 7 days. Based on syntenic analysis with *A. thaliana*, orthologues in the subgenomes were identified for comparison. Two different read alignment approaches were employed to confirm the analyses. Firstly, based on the STAR alignments as described above, genes expressed at less than 0.01 FPKM in any of the replications or for any of the orthologues were discarded. A representative set of the RNA-Seq data was assessed for unique mapping rates across the different subgenomes (Supplementary Data 16). Using three replications, for each set of orthologues genes in the hexaploid, analysis of variance was performed in R v.4.1.1^80^ to observe differences in expression patterns between the subgenomes. More than 11,000 triplicates were studied for all the samples belonging to hexaploid *Camelina* species, and the difference in the number of genes representing each category was determined using an analysis of variance test with a significant threshold of *P-*value < 0.05. Subgenome gene expression dominance analysis was also performed by comparing the relative expression of three homoeologues^46^, where the percentage of genes representing triad position above 50% are counted for all three subgenomes to plot the percentage of genes showing bias in a triad.

To confirm the sub-genome gene expression dominance and classify dominant categories among three hexaploid *Camelina* species, transcripts per million (TPM) expression of each gene among all RNA-seq samples was calculated through Salmon^81^ using three hexaploid genomes as a reference. Low-expressed homoeologous gene triads were excluded based on the sum of TPM from the SG1, SG2 and SG3 subgenome homoeologues (<1 TPM) in any RNA-seq samples. After filtering low-expressed gene triads, the relative expression (RE) of each homoeologue (SG1, SG2, SG3) was normalized using their TPM values as follows:

1 $$R{E}_{{SGn}}=\frac{{TPM}({SGn})}{{TPM}\left({SG}1\right)+{TPM}\left({SG}2\right)+\,{TPM}\left({SG}3\right)}$$

The mean RE values from all replicates were calculated as the final RE for each sample. The final RE value was used to define dominance patterns based on three categories classified in a previous study^46^; SG1 dominant when 0 ≤ RE_SG2_ ≤ 0.25 and 0 ≤ RE_SG3_ ≤ 0.25; SG2 dominant when 0 ≤ RE_SG1_ ≤ 0.25 and 0 ≤ RE_SG3_ ≤ 0.25; SG3 dominant when 0 ≤ RE_SG1_ ≤ 0.25 and 0 ≤ RE_SG2_ ≤ 0.25.

### 3D model construction for the whole genome structure

Raw Fastq files from HiC experiments were processed using the NucProcess tool^38^ to create HiC contact files. The output is generated in Nuc Chromatin Contact (NCC) format. A random set of 500,000 valid contacts was selected three times for each sample to create 3D coordinates in PDB (ProteinDataBank) format using NucDynamics tool (https://github.com/tjs23/nuc_dynamics). The 3D genome structure was viewed and expanded using PyMOL (Version 3.0, Schrödinger, LLC). Syntenic genomic blocks among the three subgenomes were extracted for contact number comparison. Only blocks with similar genomic lengths (no more than 30% length difference among the three syntenic blocks) were selected for valid contact extraction to avoid bias caused by the differing sizes of syntenic blocks. Contact numbers between SG1 and SG2, SG1 and SG3, or SG2 and SG3 were calculated separately for the comparison between different accessions.

### Genetic diversity analysis among *Camelina microcarpa* Type 2 accessions

For genetic diversity analysis, the genotyping by sequencing (GBS) data from Chaudhary et al.^10^ were used for those genotypes with 19 chromosomes. Read mapping was performed against the CmiT2 assembly, and SNP calling was performed as described in Chaudhary et al.^10^. GenAlEx v.6.5^82^ was used to determine the heterozygosity and population differentiation. STRUCTURE^83^ with a burn-in period of 150,000 steps and 150,000 MCMC was used to determine the admixture in the genotypes and the number of subpopulations, AveDissR Package in R^84^ was used for principal component analysis and MEGA X^85^ was used to draw the Neighbor-Joining tree.

### Development of interspecific segregating populations

Interspecific hybridization between *C. sativa* (CN120019)× *C. microcarpa* (CN120025) was performed; only 6 pods (1 seed) formed after manual pollination of 190 flowers. The reciprocal cross, with a different accession of *C. microcarpa* (CN119102), produced 71 seeds from 13 pods. Most of the hybrid seeds were deformed, and only 6 out of 71 seeds germinated. *Camelina microcarpa* was a winter-type and required vernalization to reach the reproductive phase, whereas CN120019 (*C. sativa*) was a spring-type. The hybrids from these crosses produced a winter-type plant that had an intermediate plant and leaf morphology from the parents. The CN119102 × CN120019 cross produced four F_1_ plants, although partially sterile, together they produced 49 F_2_ seeds. One of the hybrids was backcrossed with accession PI650132, a spring-type *C. alyssum* to generate BC_1_F_1_ plants. A total of 8 seeds were obtained from the (*C. microcarpa* × *C. sativa*) × *C. alyssum* cross, and one plant was used to generate the BC_1_F_2_ generation. BC_1_F_2_ plants derived from selfed-seed from the (CN119102 × CN120019) × PI650132 cross were used for genetic analysis.

### Genotyping of segregating lines

One-week-old leaf tissue was harvested and kept at −80 °C until DNA extraction. DNA extractions were performed using the CTAB method, and GBS library preparation was as described by Poland et al.^86^. Briefly, after DNA normalization, 200 ng of DNA were digested with *PstI* and *MspI*, adapters were ligated to the restriction-digested DNA fragments, before multiplexing and pooling of 96 samples into a single library. After pooling, the library was amplified, purified and quantified using a Bioanalyzer (Agilent Technologies) to confirm fragment size and quality. Paired-end sequencing was done on the Illumina HiSeq platform. Sequences were trimmed for low base quality and adapters using Trimmomatic v.0.33^60^. Reads were mapped using BWA^70^, with default parameters, to a pseudo-genome generated from combining the CsaDH55 genome with the third subgenome from CmiT2, making a pseudogenome with four subgenomes (26 chromosomes). SNPs were called using GATK^87^ with default parameters. Sequence reads were used to study possible aneuploidy and homoeologous recombination. BEDTools^88^ was used to extract mapped reads and to calculate the frequency of mapped reads along 100 Kb bins in the genome. Reads were plotted across the bins to confirm possible genetic events. Circos^89^ and karyoploteR^90^ were used to plot the distribution of mapped reads along the *Camelina* pseudogenome for visualizing aneuploidy and homoeologous recombination events.

### Chloroplast assembly and analysis

Chloroplast genomes of CsaDH55, CmiT1, Cmi4X, and four *C. sativa* genotypes (MAGIC05, MAGIC08, MAGIC10 and MAGIC15) were assembled from PacBio long read data using Oatk^91^, whereas the chloroplast for CmiT2 was assembled using GetOrganelle^92^ using short Illumina reads. The chloroplast assembly for *C. neglecta*, *C. laxa*, *C. hispida* were adopted from Martin et al.^23^. All the assembled chloroplast genomes were aligned using Clustal Omega^93^ and visualized using MEGA11^94^.

### Reporting summary

Further information on research design is available in the Nature Portfolio Reporting Summary linked to this article.

## Supplementary information

Supplementary Information

Peer Review file

Description of Additional Supplementary Files

Supplementary Data 1

Supplementary Data 2

Supplementary Data 3

Supplementary Data 4

Supplementary Data 5

Supplementary Data 6

Supplementary Data 7

Supplementary Data 8

Supplementary Data 9

Supplementary Data 10

Supplementary Data 11

Supplementary Data 12

Supplementary Data 13

Supplementary Data 14

Supplementary Data 15

Supplementary Data 16

Reporting Summary

## Source data

Source Data

## Data Availability

All new sequence data is deposited at the EBI-ENA under accession PRJEB96055. The genome assemblies and annotations are available at Crucifer Genome Initiative database (https://cruciferseq.ca). Short-read *C. sativa* DH55 genomic data from Kagale et al.^13^ was used: SRR1171872 and SRR1171873. Transcriptome data from twelve tissue types of *C. sativa* DH55 was used^14^: germinating seed SRX472932, cotyledons SRX472934, young leaf SRX472935, senescing leaf SRX472936, stem SRX472937, root SRX472938, buds SRX472939, flower SRX472941, early seed development SRX472942, early mid seed development SRX472943, late mid seed development SRX472945 and late seed development SRX472946. Source data are provided with this paper.
