# Supplementary material for: A fourth subgenome of the Camelina genus reveals gene dominance is influenced by chromosomal proximity
Source: Nat Commun. 2026 Jun 26;17:7992. doi: 10.1038/s41467-026-74800-8 (PMC13448894; doi:10.1038/s41467-026-74800-8)
Supplement: Supplementary file 1 [file 41467_2026_74800_MOESM1_ESM.pdf]

A fourth subgenome of the *Camelina* genus reveals gene dominance is influenced by chromosomal proximity

Chaudhary *et al.*

Supplementary Table 1. Sequencing technologies and amount of data utilized for the genome assemblies.

Genotype	Genome size (Gb)	Coverage (x)				Sequencing platforms
		Oxford Nanopore Technology	Pacific Bioscience	HiC	Illumina	
**Cmi4X (CN119243)**	0.441	56.7	33.3	227.2	123.8	GridIon, Sequel IIe, NovaSeq 6000
**CmiT1 (CN119205)**	0.729	47.5	13.9	131.1	33.5	GridIon, Sequel IIe, NovaSeq 6000
**CmiT2 (CN120025)**	0.634	106.8	25.9	84.3	65.3	PromethION 2 Solo, PacBio Sequel I, NovaSeq 6000
**CsaDH55 (DH55)**	0.75	12.4	36.1	164.0	NA*	MInIOn, Sequel IIe, NovaSeq 6000

*Illumina reads for DH55 (SRR1171872 & SRR1171873) were used from Kagale *et al.*[1].

Supplementary Table 2. Statistics of Illumina reads mapped to the respective assembled genomes.

Parameter	Cmi4X (CN119243)	CmiT2 (CN120025)	CmiT1 (CN119205)	DH55*
Number of reads (Million)	392	252	170	183
Mapped reads (%)	99.28	99.48	99.42	99.86
general error rate	0.0029	0.0049	0.0027	0.12
Duplication rate (%)	39.48	19.80	32.35	16.79
GC Percentage (%)	35.41	35.30	36.34	35.98
Mean coverage	124.41	53.84	39.98	26.75

*Illumina read for DH55 (SRR1171872 & SRR1171873) were used from Kagale *et al.*[1].

Supplementary Table 3. Tentative centromeric position for Cmi4X (CN119243), CmiT1 (CN119205), CmiT2 (CN120025), and CsaDH55 (Cs_genome_v3)

Cmi4X (CN119243)	Putative centromere			Ancestral Centromere		
	Centromere start	Centromere end	Centromere size	Centromere start	Centromere end	Centromere size
Chr01	19,300,001	19,500,000	199,999			
Chr02	10,300,001	11,000,000	699,999			
Chr03	16,200,001	16,400,000	199,999	19,700,001	19,900,000	199,999
Chr04	7,800,001	8,000,000	199,999	10,000,001	10,200,000	199,999
Chr05	13,000,001	13,500,000	499,999			
Chr06	19,700,001	20,000,000	299,999			
Chr07	17,500,001	18,200,000	699,999			
Chr08	10,800,001	11,000,000	199,999			
Chr09	18,300,001	20,600,000	2,299,999			
Chr10	7,800,001	9,200,000	1,399,999			
Chr11	14,700,001	14,900,000	199,999			
Chr12	16,500,001	16,800,000	299,999			
Chr13	6,900,001	7,100,000	199,999			

CmiT2 (CN120025)	Putative centromere		
	Centromere start	Centromere end	Centromere size
Chr1	17,200,000	20,300,000	3,100,000
Chr2	10,200,000	11,000,000	800,000
Chr3	18,800,000	19,900,000	1,100,000
Chr4	8,100,000	9,300,000	1,200,000
Chr5	12,900,000	13,500,000	600,000
Chr6	11,000,000	11,900,000	900,000
Chr7	15,500,000	17,600,000	2,100,000
Chr8	10,800,000	12,800,000	2,000,000
Chr9	14,300,000	15,000,000	700,000
Chr10	6,500,000	7,600,000	1,100,000
Chr11	14,400,000	17,000,000	2,600,000
Chr12	15,400,000	16,700,000	1,300,000
Chr13	5,800,000	6,900,000	1,100,000
Chr14	20,300,000	21,800,000	1,500,000
Chr15	12,600,000	14,200,000	1,600,000
Chr16	25,400,000	26,500,000	1,100,000
Chr17	8,800,000	10,900,000	2,100,000
Chr18	27,500,000	28,000,000	500,000
Chr19	12,800,000	13,900,000	1,100,000

CmiT1 (CN119205)	Putative centromere			Ancestral Centromere		
	Centromere start	Centromere end	Centromere size	Centromere start	Centromere end	Centromere size
Chr01	19,200,001	19,300,000	99,999			
Chr02	10,300,001	10,900,000	599,999			
Chr03	19,800,001	20,600,000	799,999	16,100,001	16,300,000	199,999
Chr04	9,300,001	10,200,000	899,999			
Chr05	13,000,001	13,600,000	599,999			
Chr06	19,500,001	19,800,000	299,999	27,800,001	28,000,000	199,999
Chr07	17,600,001	18,500,000	899,999			
Chr08	10,800,001	11,000,000	199,999			
Chr09	18,300,001	20,000,000	1,699,999			
Chr10	7,600,001	8,600,000	999,999			
Chr11	14,500,001	15,900,000	1,399,999			
Chr12	16,600,001	16,800,000	199,999			
Chr13	6,800,001	8,200,000	1,399,999			
Chr14	20,700,001	21,000,000	299,999			
Chr15	22,800,001	22,900,000	99,999			
Chr16	19,400,001	19,600,000	199,999			
Chr17	10,700,001	11,600,000	899,999	13,800,001	14,000,000	199,999
Chr18	19,000,001	19,300,000	299,999	20,900,001	21,100,000	199,999
Chr19	11,300,001	11,400,000	99,999			
Chr20	22,700,001	22,900,000	199,999			

Cs_genome_v3	Putative centromere			Ancestral Centromere		
	Centromere start	Centromere end	Centromere size	Centromere start	Centromere end	Centromere size
Chr01	18,600,001	22,400,001	3,800,000			
Chr02	12,800,001	14,800,001	2,000,000			
Chr03	18,000,001	22,300,001	4,300,000			
Chr04	9,300,001	12,500,001	3,200,000			
Chr05	23,200,001	25,900,001	2,700,000			
Chr06	7,900,001	10,600,001	2,700,000			
Chr07	10,500,001	12,600,001	2,100,000			
Chr08	14,200,001	14,800,001	600,000			
Chr09	12,000,001	14,100,001	2,100,000	17,000,001	17,300,001	300,000
Chr10	16,700,001	18,800,001	2,100,000			
Chr11	19,600,001	22,600,001	3,000,000			
Chr12	22,900,001	26,300,001	3,400,000			
Chr13	16,200,001	18,200,001	2,000,000	14,500,000	14,600,000	100,000
Chr14	19,300,001	23,500,001	4,200,000			
Chr15	20,400,001	22,300,001	1,900,000	17,900,001	18,200,001	300,000
Chr16	11,000,001	14,000,001	3,000,000			
Chr17	18,900,001	21,400,001	2,500,000	23,500,001	23,900,001	400,000
Chr18	7,500,001	10,000,001	2,500,000			
Chr19	19,800,001	22,400,001	2,600,000			
Chr20	18,600,001	20,700,001	2,100,000			

Supplementary Table 4. Number of syntenic genes retained in different *Camelina s*pecies relative to *A. thaliana.*

**Genome**	**Number of genes**	**Percentage**
C. neglecta	21,335	93.40
SG1_Cmi4X	20,598	90.17
SG2_Cmi4X	20,529	89.87
SG1_CmiT1	20,544	89.94
SG2_CmiT1	20,502	89.75
SG3_CmiT1	20,537	89.91
SG1_CmiT2	20,452	89.53
SG2_CmiT2	20,453	89.54
SG3_CmiT2	20,564	90.02
SG1_CsaDH55	20,543	89.93
SG2_CsaDH55	20,505	89.76
SG3_CsaDH55	19,816	86.75
C. hispida	20,385	89.24

Supplementary Table 5. Number of RGA genes represented in clusters across the different *Camelina* genomes.

**Number of RGA genes in a cluster**	***C. neglecta***	**Cmi4X (CN119243)**	**CmiT2 (CN120025)**	**CmiT1 (CN119205)**	**Cs_genome_v3**
1	405	829	1174	1268	1292
2	89	182	283	275	257
3	24	66	111	115	110
4	11	48	56	50	69
5	6	15	28	27	24
6	7	15	23	31	25
7	5	10	14	17	22
8	3	4	6	5	4
9	2	6	7	8	5
10 or more	5	18	29	28	24

Supplementary Table 6. Number of significant (q-value<0.01) intra- and inter-chromosomal interactions for each of the *Camelina* species.

Interaction	Cmi4X (CN119243)	CmiT2 (CN120025)	CmiT1 (CN119205)	CsaDH55
**Intra-chromosomal**	616,348	1,858,814	888,946	134,060
**Inter-chromosomal**	67,735	231,297	109,414	13,481
**Total**	**684,083**	**2,090,111**	**998,360**	**147,541**
***Subgenome interaction calculated from omni-c data fithic tool**				
**SG1-SG2**	48,839	63,548	32,700	3,302
**SG1-SG3**		64,631	27,798	3,047
**SG2-SG3**		33,977	27,665	2,753
**Total**		**162,156**	**88,163**	**9,102**
**Subgenome interaction calculated from omni-c data using nuc_processing tool**				
**SG1-SG2**		182,116	279,311	87,589
**SG1-SG3**		231,222	165,389	63,661
**SG2-SG3**		129,386	154,446	66,841
**Total**		**542,724**	**599,146**	**218,091**
**Subgenome interaction calculated from proximo HiC data using nuc_processing tools**				
**SG1-SG2**		102,898	218,256	22,048
**SG1-SG3**		120,447	173,314	19,802
**SG2-SG3**		77,141	175,654	18,070
**Total**		**300,486**	**567,224**	**59,920**

*Note: Bins with significant interactions >1000 were removed

Supplementary Table 7. Genetic diversity parameters among 18 genotypes belonging to *Camelina microcarpa* T2 genome.

Pop		Na	Ne	I	Ho	He	uHe	F
**Pop1**	**Mean**	1.608	1.296	0.286	0.006	0.184	0.193	0.958
	**SE**	0.010	0.006	0.005	0.000	0.003	0.004	0.003
**Pop2**	**Mean**	1.525	1.300	0.275	0.010	0.181	0.196	0.942
	**SE**	0.010	0.007	0.005	0.001	0.004	0.004	0.003
**Total**	**Mean**	1.566	1.298	0.280	0.008	0.183	0.194	0.951
	**SE**	0.007	0.005	0.004	0.000	0.003	0.003	0.002

Total	Fis	Fit	Fst
**Mean**	0.961	0.973	0.221
**SE**	0.002	0.002	0.005

Na = No. of Different Alleles
Ne = No. of Effective Alleles = 1/(Sum pi^2)
I = Shannon's Information Index = -1* Sum (pi * Ln (pi))
Ho = Observed Heterozygosity = No. of Hets/N
He = Expected Heterozygosity = 1 - Sum pi^2
uHe = Unbiased Expected Heterozygosity = (2N/(2N-1)) * He
F = Fixation Index = (He - Ho)/He = 1 - (Ho/He)
Where pi is the frequency of the ith allele for the population & Sum pi^2 is the sum of the squared population allele frequencies.

Fis = (Mean He - Mean Ho)/Mean He
Fit = (Ht - Mean Ho)/Ht
Fst = (Ht - Mean He)/Ht

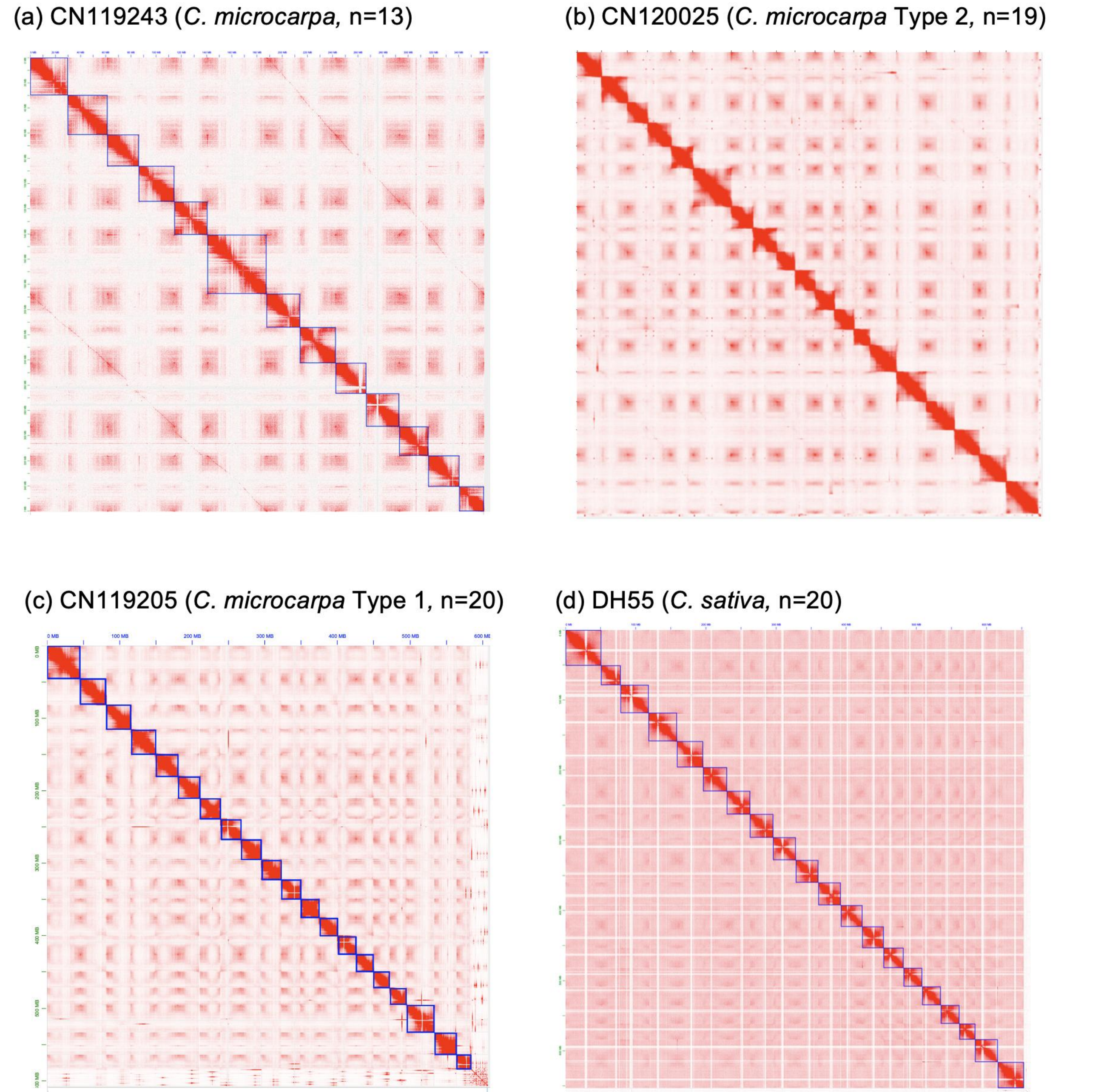

Supplementary Figure 1. Scaffolding of *Camelina* genomes using HiC technology. HiC contact maps for a) CN119243, b) CN120025, c) CN119205, and d) DH55. Dark red regions along the diagonal indicates strong intra chromosomal interaction, while clear boundaries between chromosomes are indicated with blue frames.

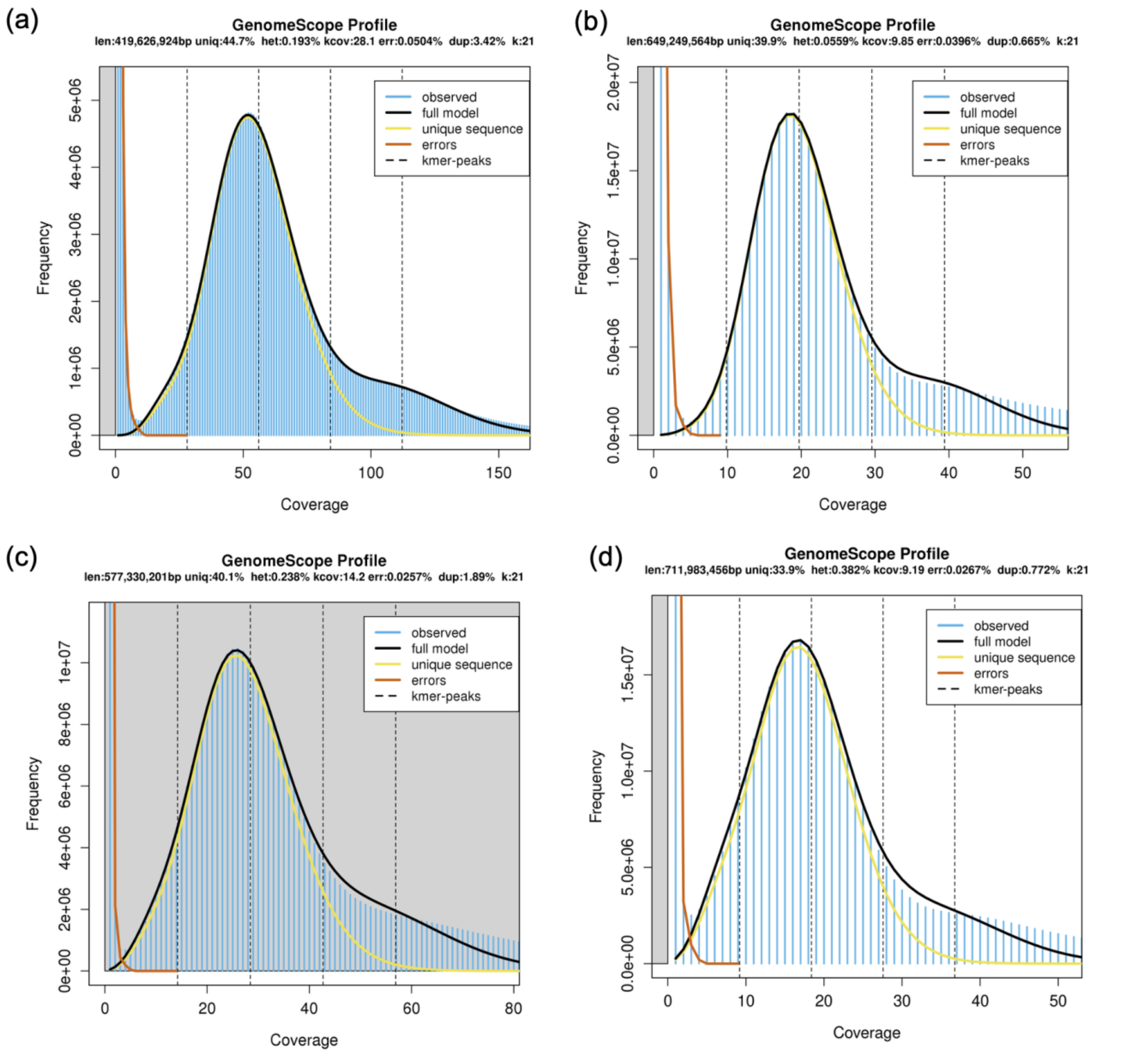

Supplementary Figure 2. **Genome size estimation in *Camelina* species using kmer analysis.** Four new genomes of *Camelina* species - *C. microcarpa* Tetraploid (a), *C. microcarpa* T1 (b), *C. microcarpa* T2 (c), and DH55 (d).

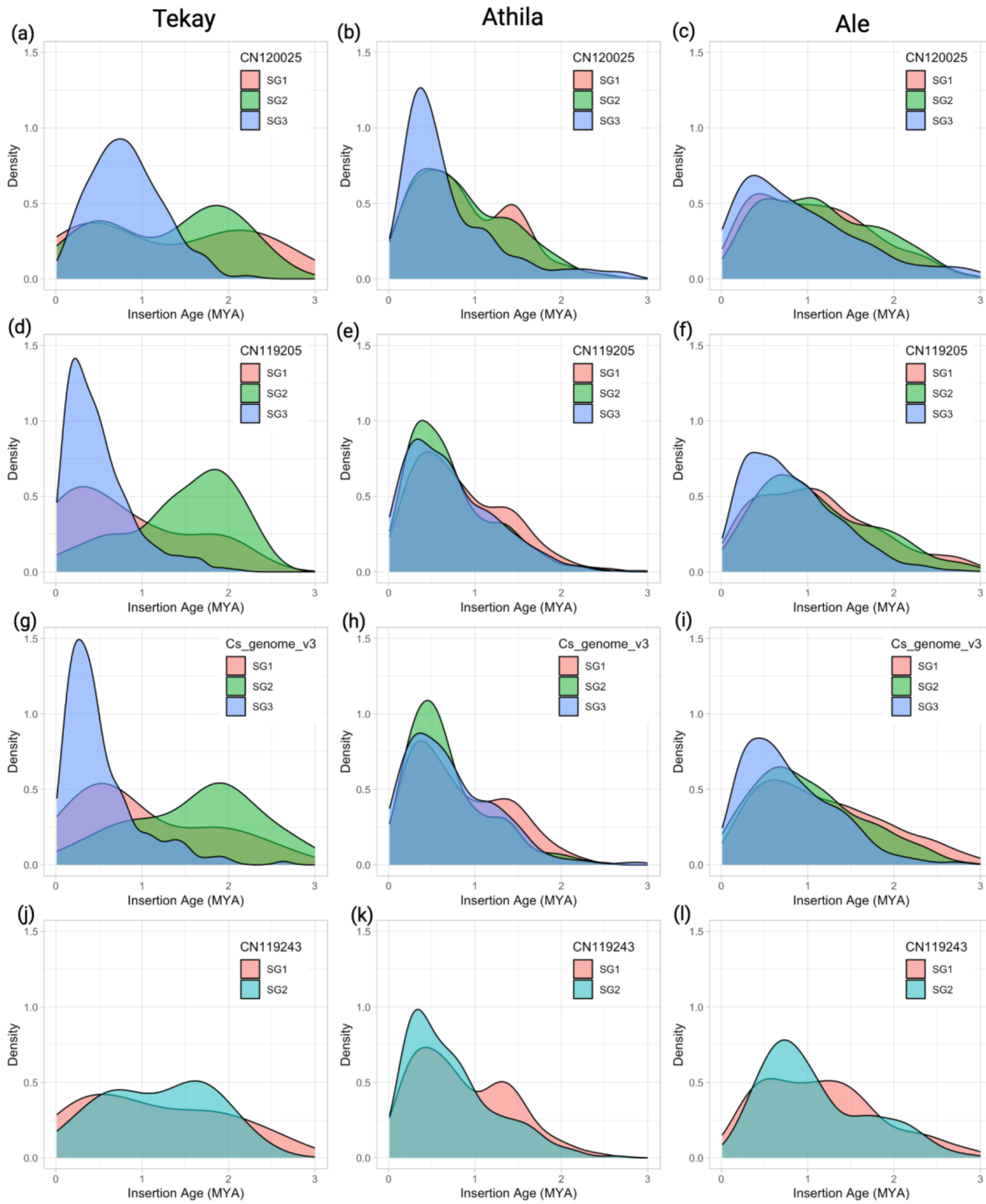

Supplementary Figure 3. **Density of TE elements across hexaploid *Camelina* species**. The specific element represented in each genome are: a) Tekay CmiT2, b) Athila CmiT2, c) Ale CmiT2, d) Tekay CmiT1, e) Athila CmiT1, f) Ale CmiT1, g) Tekay CsaDH55, h) Athila CsaDH55, i) Ale CsaDH55, j) Tekay Cmi4X, k) Athila Cmi4X, and l) Ale Cmi4X. CmiT1 is a *C. microcarpa* T1; CmiT2 is a *C. microcarpa* T2, Cmi4X is a tetraploid *C. microcarpa*, and CsaDH55 is *C. sativa*.

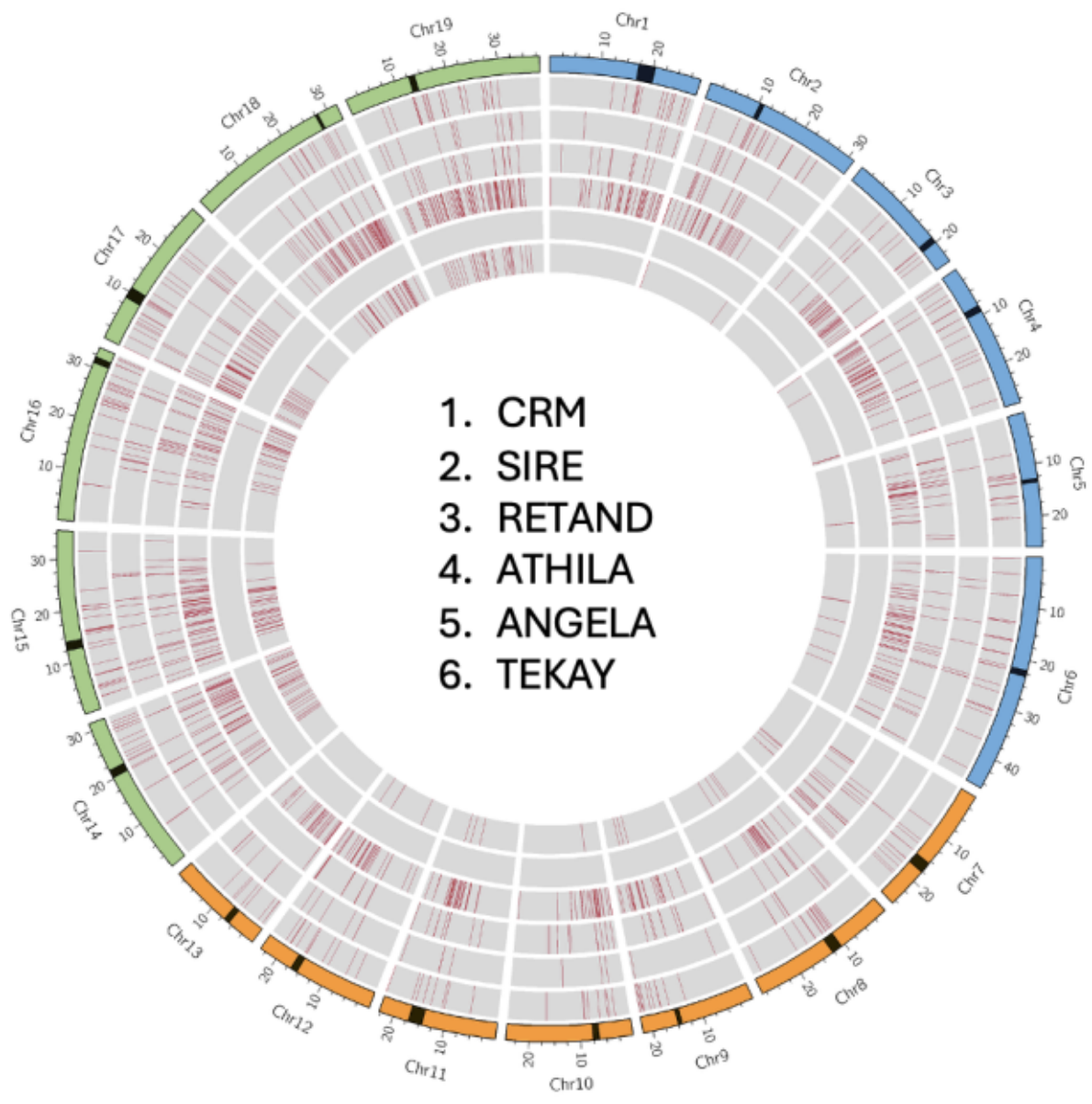

(a) CmiT2

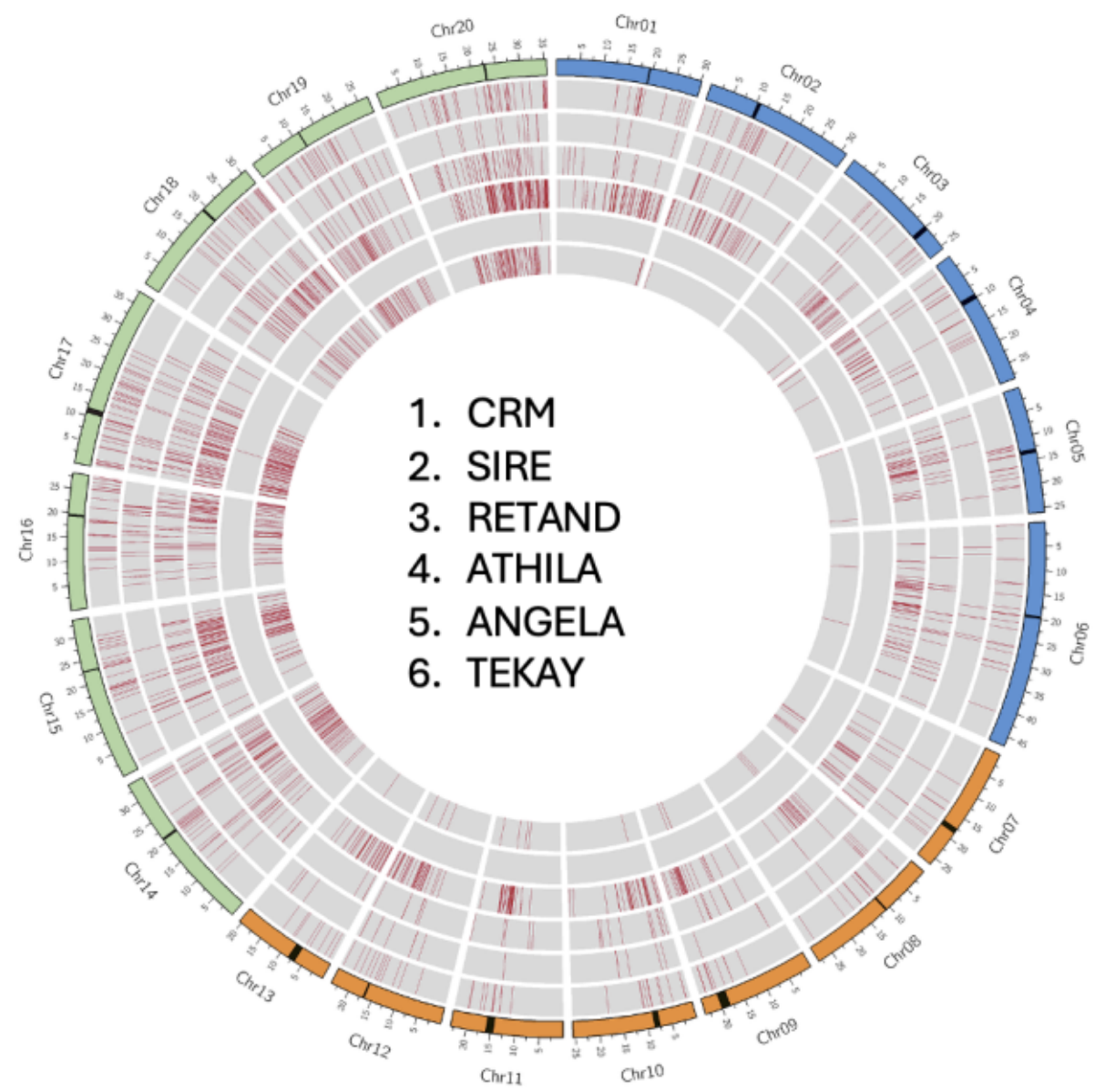

(b) CmiT1

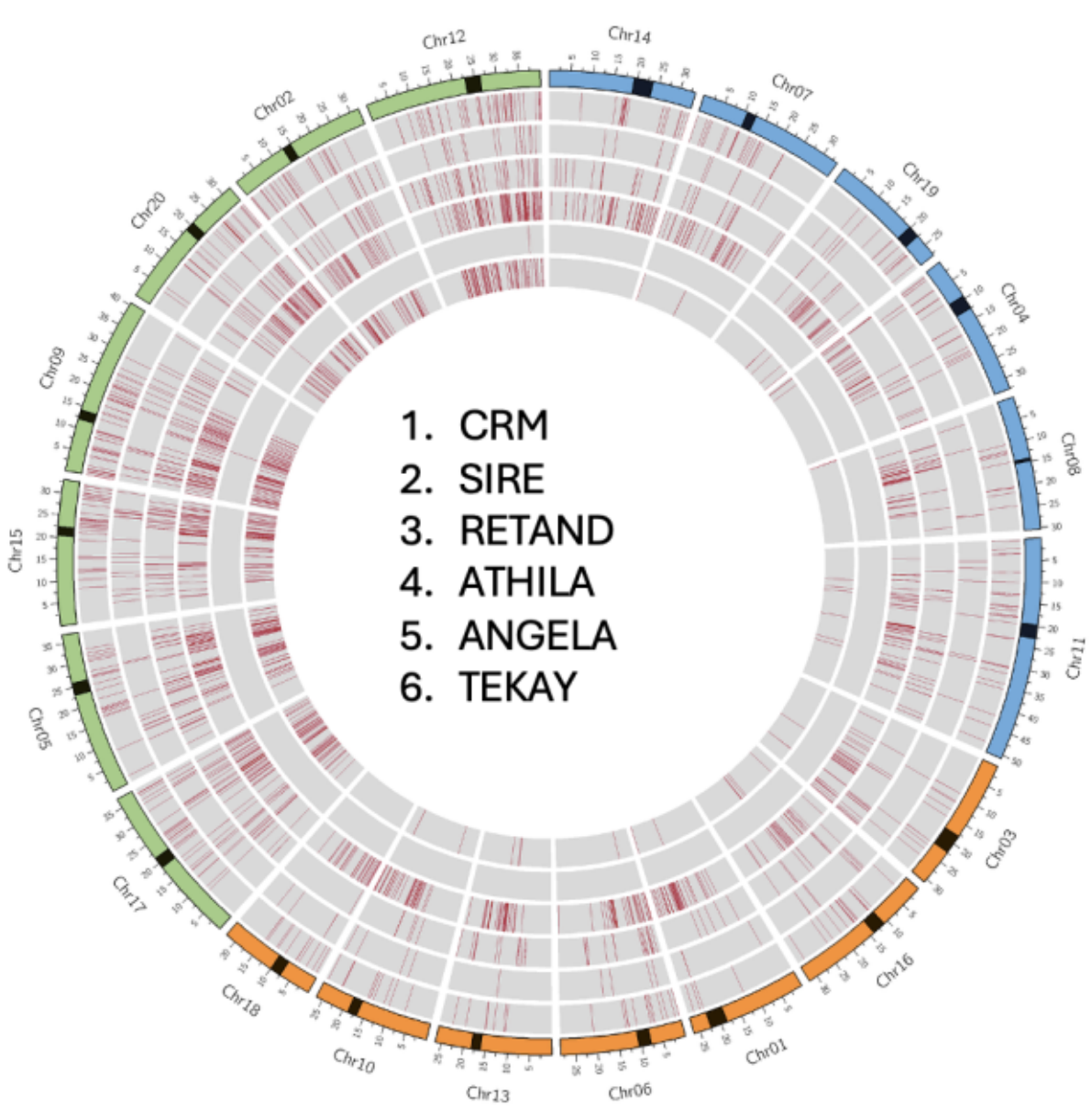

(c) CsaDH55

Supplementary Figure 4. Distribution of full length LTR transposable elements (TEs) across *Camelina* species. Full length LTR TEs across the three hexaploids are shown: (a) CmiT2; (b) CmiT1, and (c) CsaDH55. The different named LTR elements are shown in tracks 1-6 from the outside to the inside of the circos plot and indicated in the central legend.

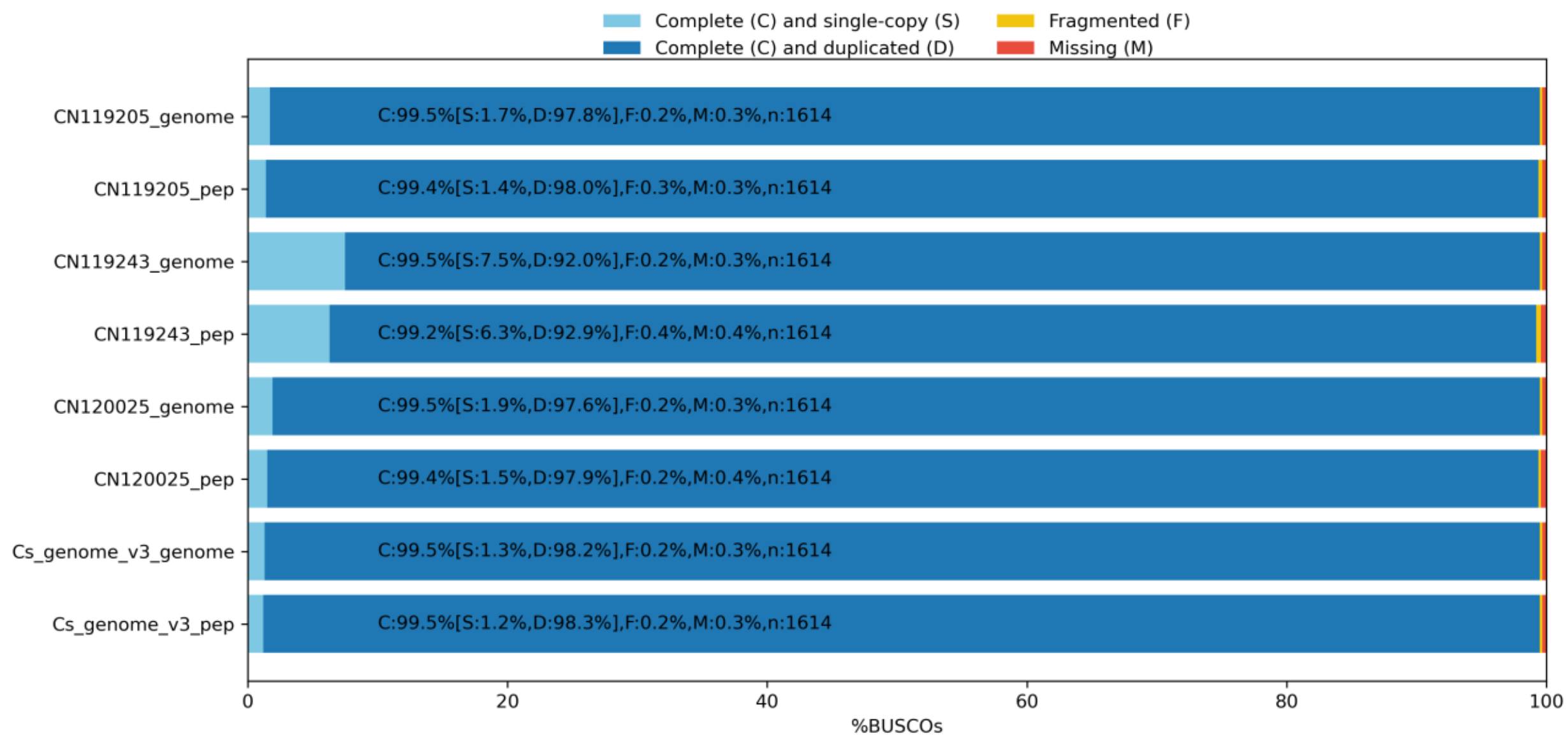

Supplementary Figure 5. **BUSCO analysis for different *Camelina* species**. CN119243 is Cmi4X, CN119205 is CmiT1, CN120025 is CmiT2, and Cs_genome_v3 is CsaDH55. The results run in both genome mode (genome assembly) and protein mode (predicted proteins) are shown.

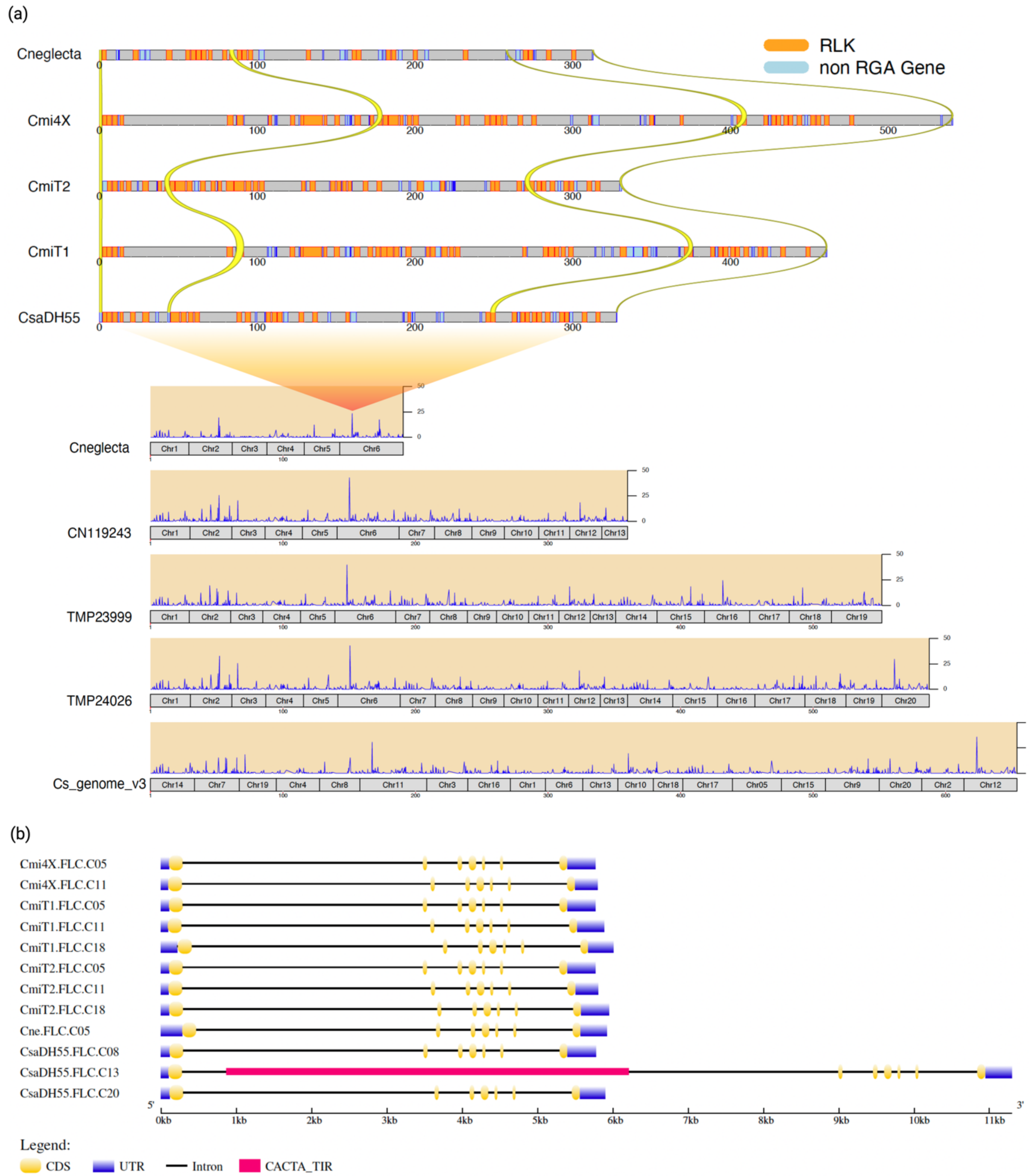

Supplementary Figure 6. Evolution of resistance genes and flowering gene in *Camelina* species. (a) RGA clustering in *Camelina* species in bottom panel with RGA density plotted in blue, the inset at top shows conservation of an RGA cluster; and (b) Conservation of *FLC* structure in *Camelina* species with a divergent *Csa.FLC.C13* structure shown by an insertion of a transposon (red). CmiT1 is a *C. microcarpa* T1; CmiT2 is a *C. microcarpa* T2, Cmi4X is a tetraploid *C. microcarpa*, and CsaDH55 is *C. sativa*.

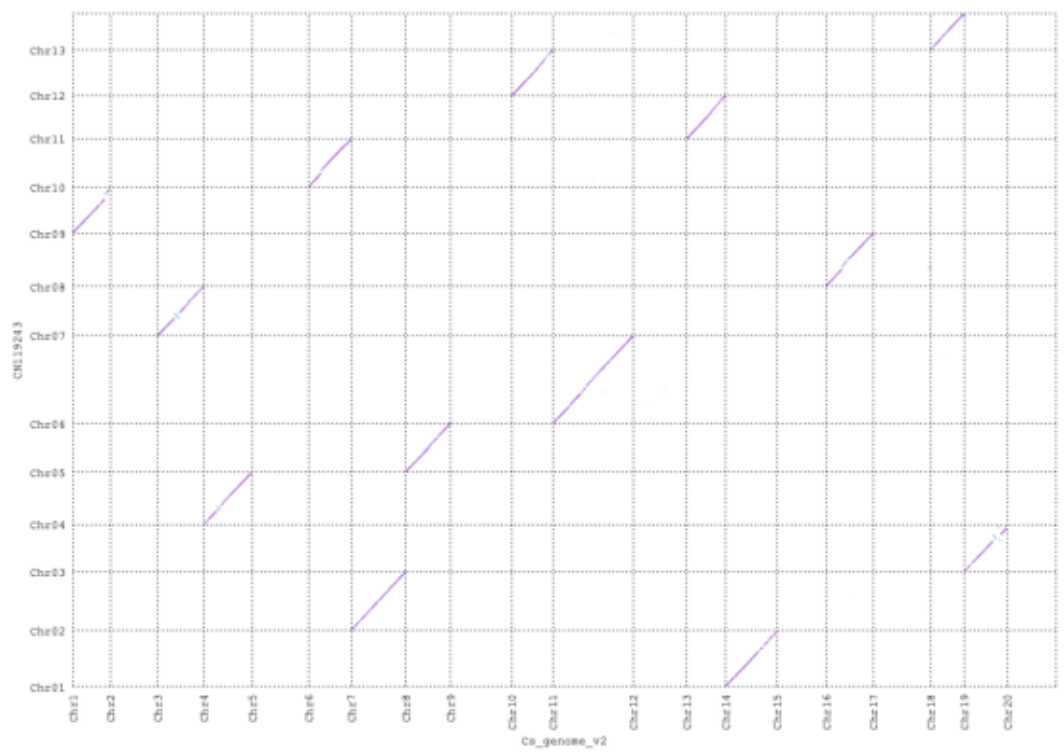

(a) DH55 - CN119243

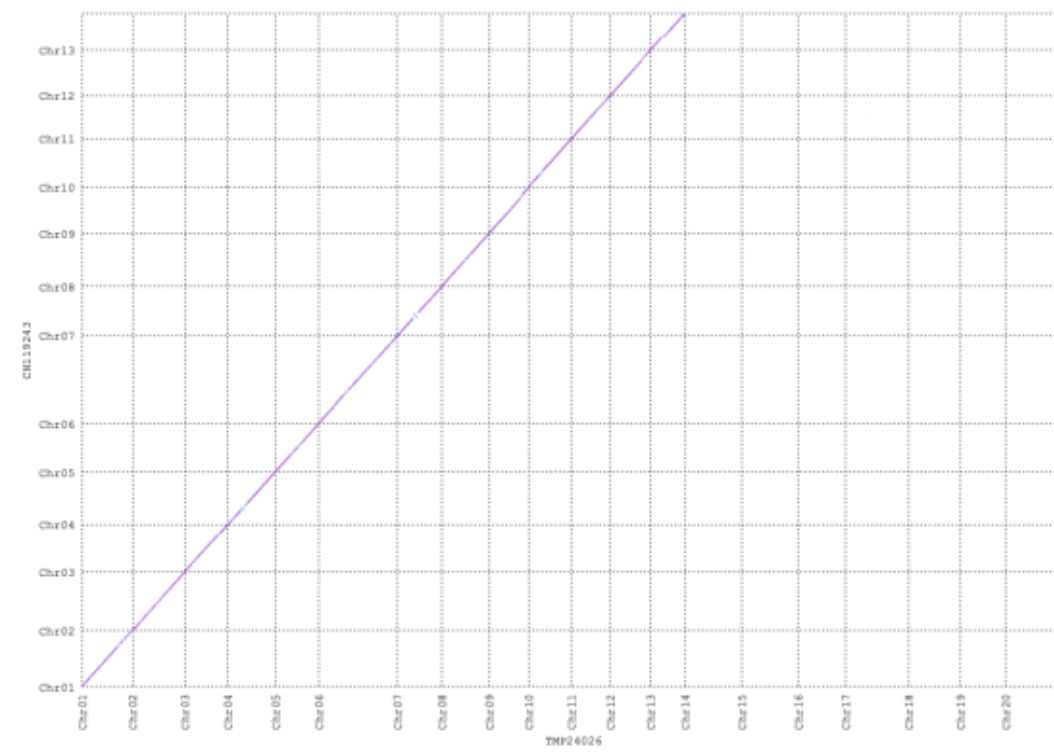

(b) CN119205 - CN119243

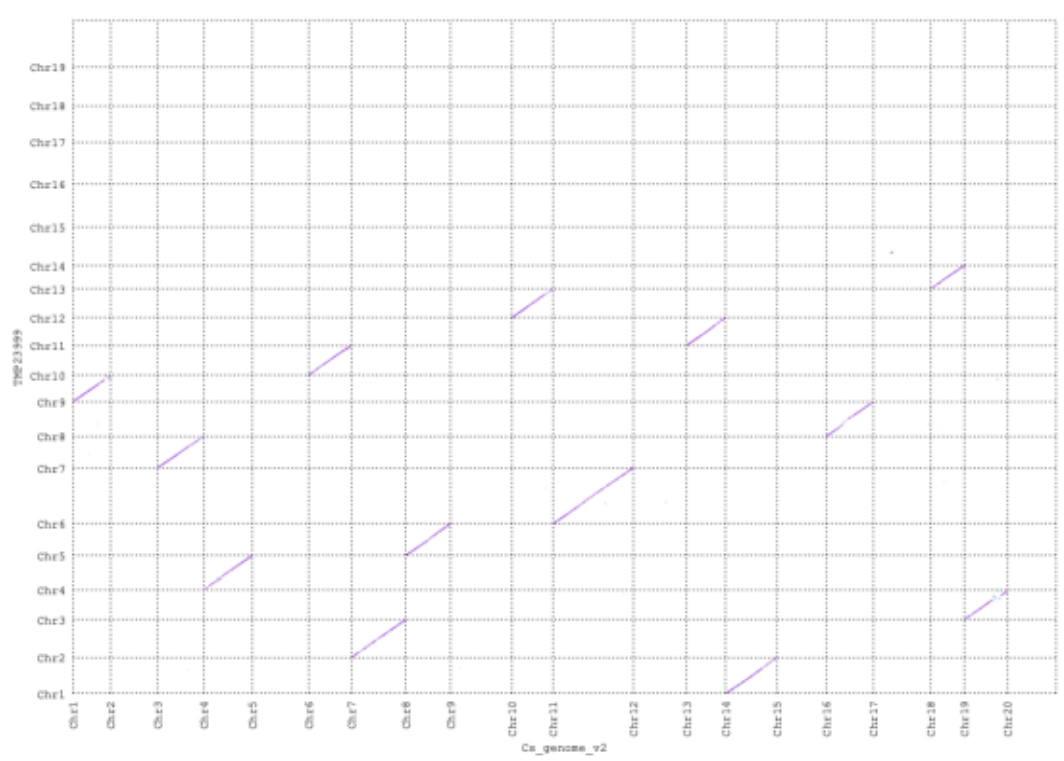

(c) DH55 - CN120025

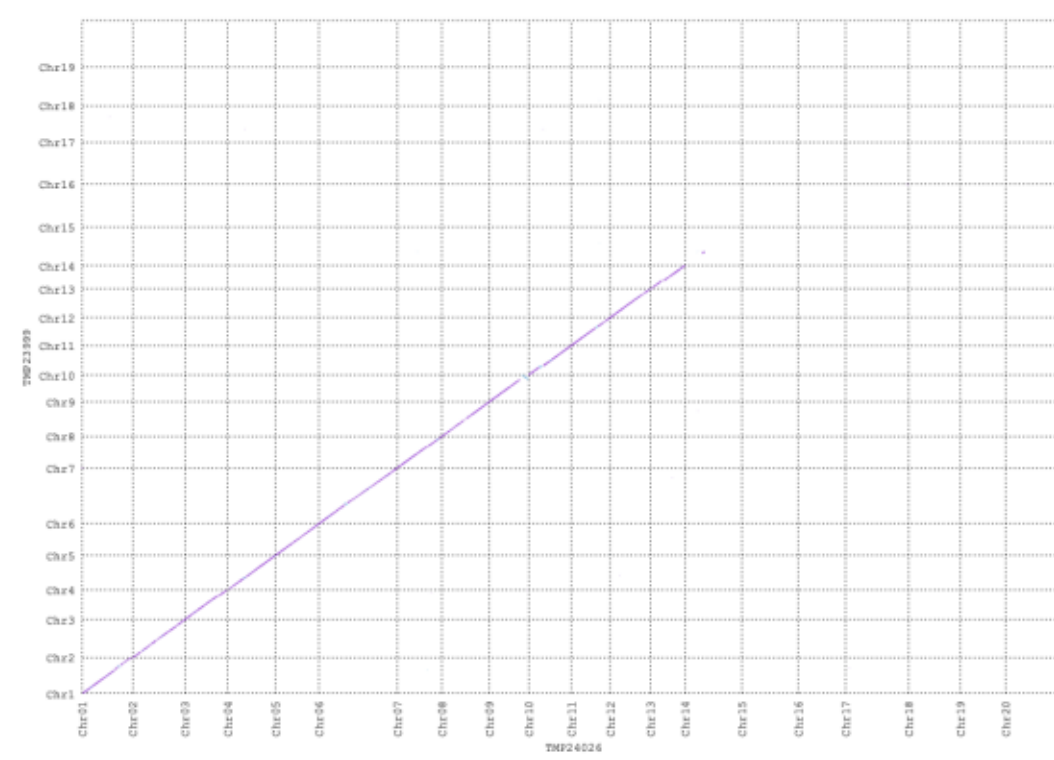

(d) CN119205 - CN120025

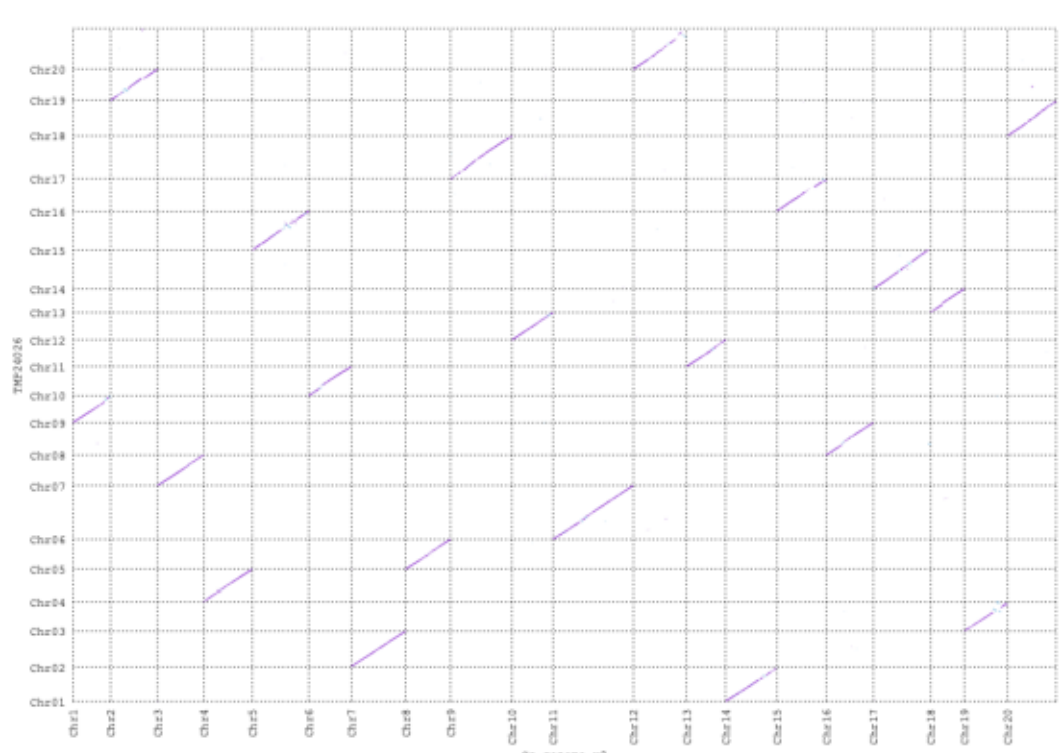

(e) DH55 - CN119205

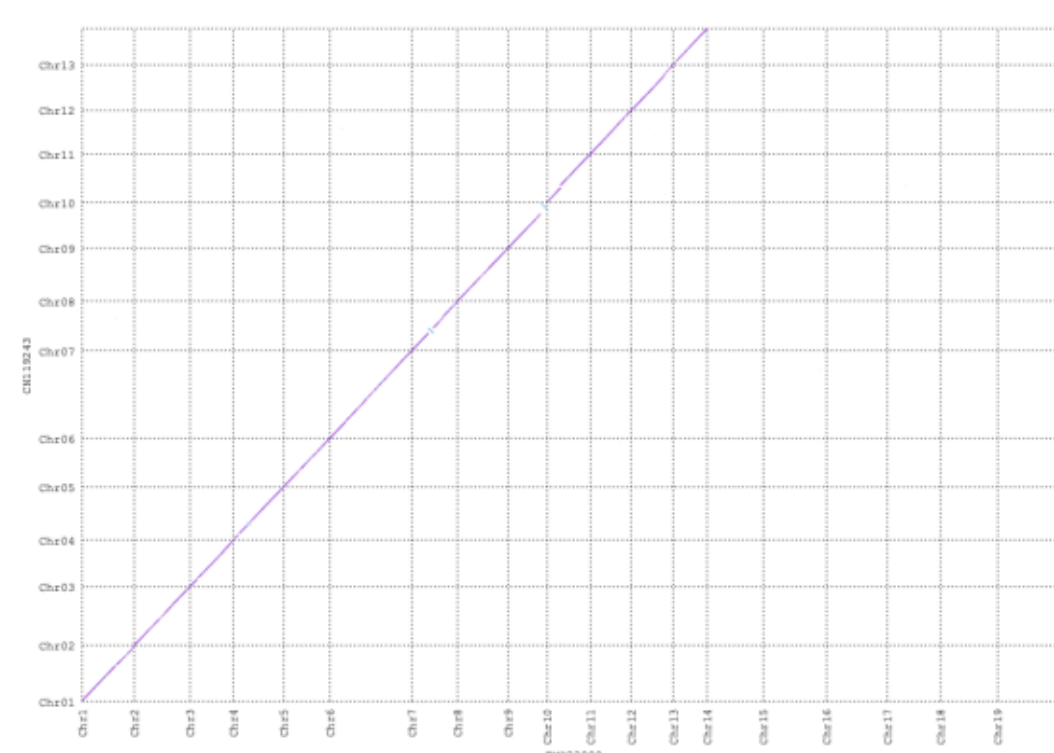

(f) CN120025 - CN119243

Supplementary Figure 7. **Relationship among different species as explained by nucmer plots**. The first genome is on the x-axis and the second on the y-axis: a) DH55 vs Cmi4X; b) Cmi4X vs CmiT1; c) DH55 vs CmiT2, d) CmiT1 vs CmiT2, e) DH55 vs CmiT1, and f) CmiT2 vs Cmi4X. CN119243 is Cmi4X, CN119205 is CmiT1, CN120025 is CmiT2, and DH55 is CsaDH55.

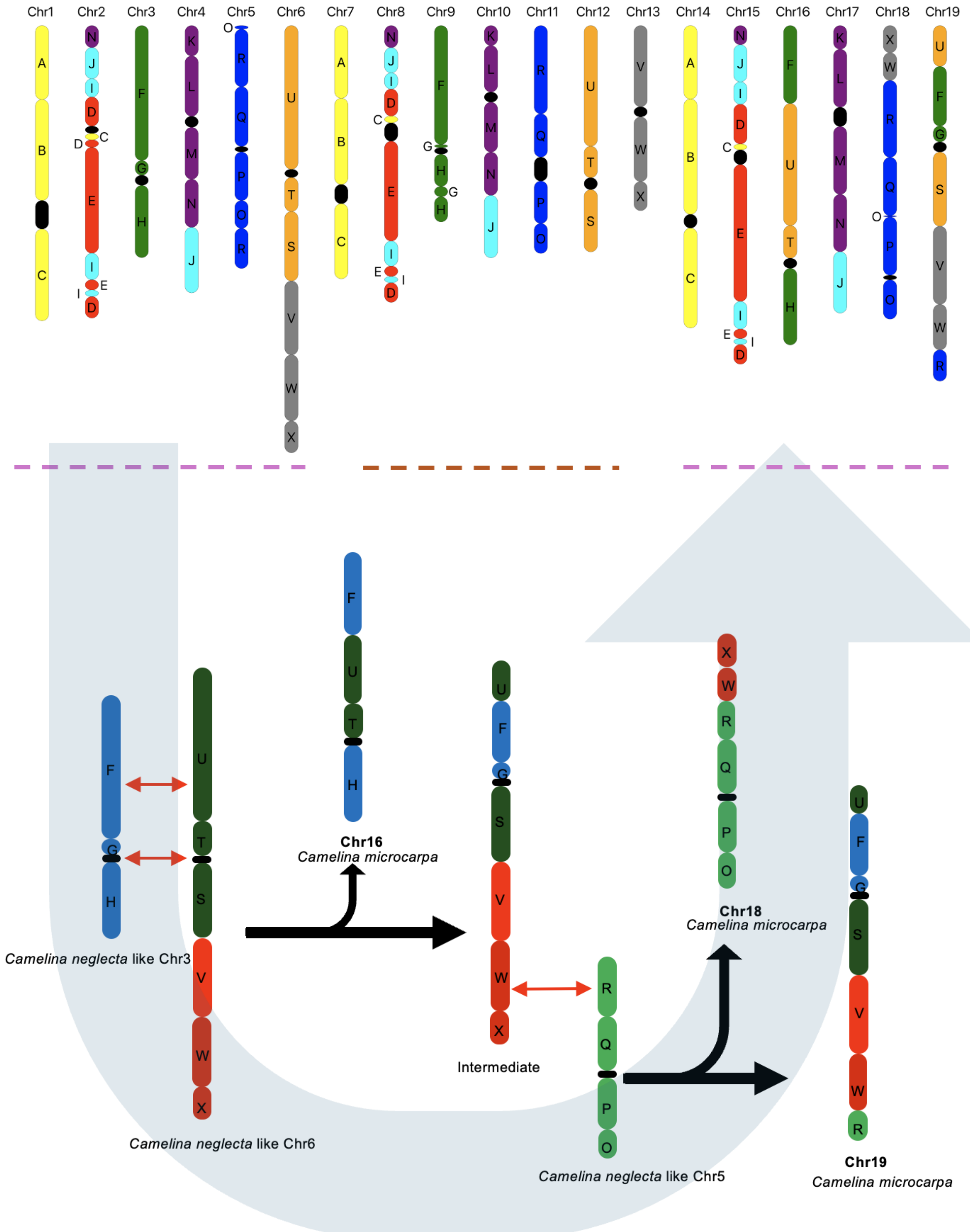

Supplementary Figure 8. **Karyotype evolution in *C. microcarpa* Type 2.** The karyotype of CmiT2 drawn from the ancestral chromosome blocks is shown at the top, while the inferred rearrangements necessary to generate the SG3 structure from a structure presumed to be similar to SG1 is shown at the bottom.

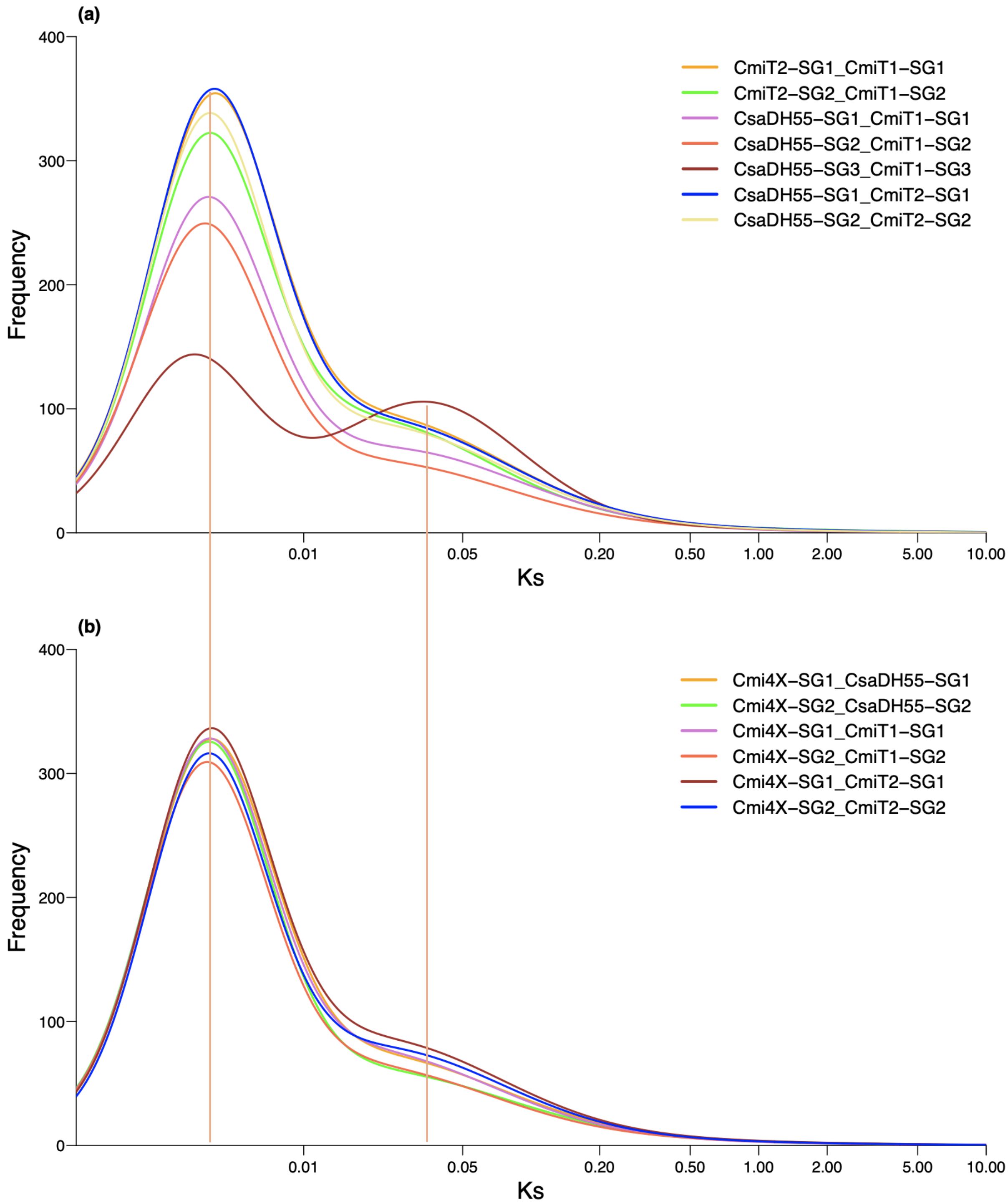

Supplementary Figure 9. **Ks Analysis of subgenomes of *Camelina* species.** Ks analysis of hexaploid species (a) and Ks analysis of tetraploid *C. microcarpa* with subgenomes of hexaploid *Camelina* species (b). CN119243 is Cmi4X, CN119205 is CmiT1, CN120025 is CmiT2, and CsaDH55 is *Camelina sativa*.

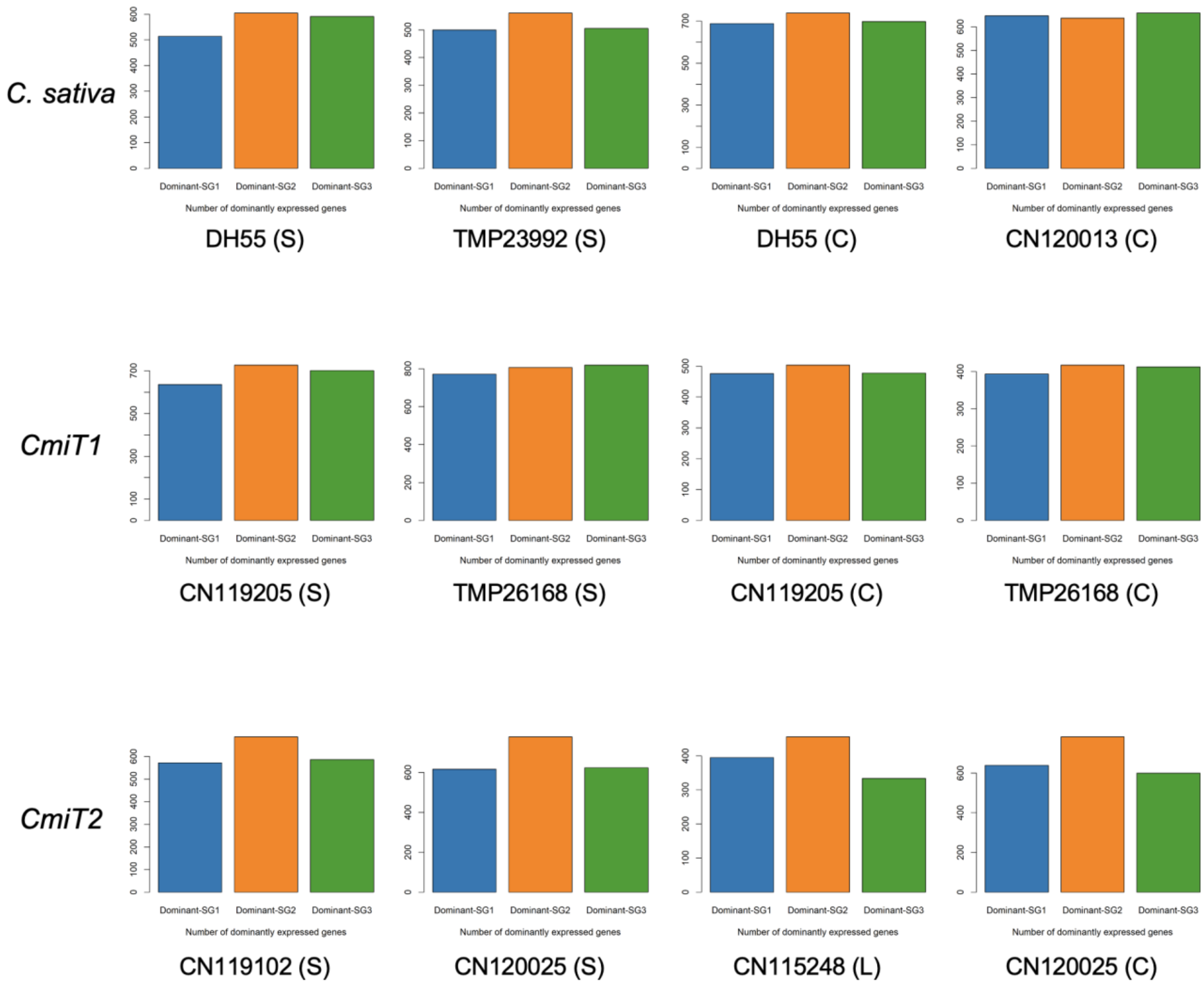

Supplementary Figure 10. Numbers of dominantly expressed genes across subgenomes in different *Camelina* hexaploids. Multiple accessions are shown for *Camelina sativa* (n=20 chromosomes), *C. microcarpa T1* (n=20), and *C. microcarpa* T2 (n=19 chromosomes) genomes. Numbers of dominantly expressed genes were calculated based on TPM values derived from Salmon read alignment of RNA-seq data. Tissues used for RNA-seq are labeled in the brackets following accession names (S: etiolated seedling; C: cotyledon; L: first true leaf). Source data are provided as a Source Data file.

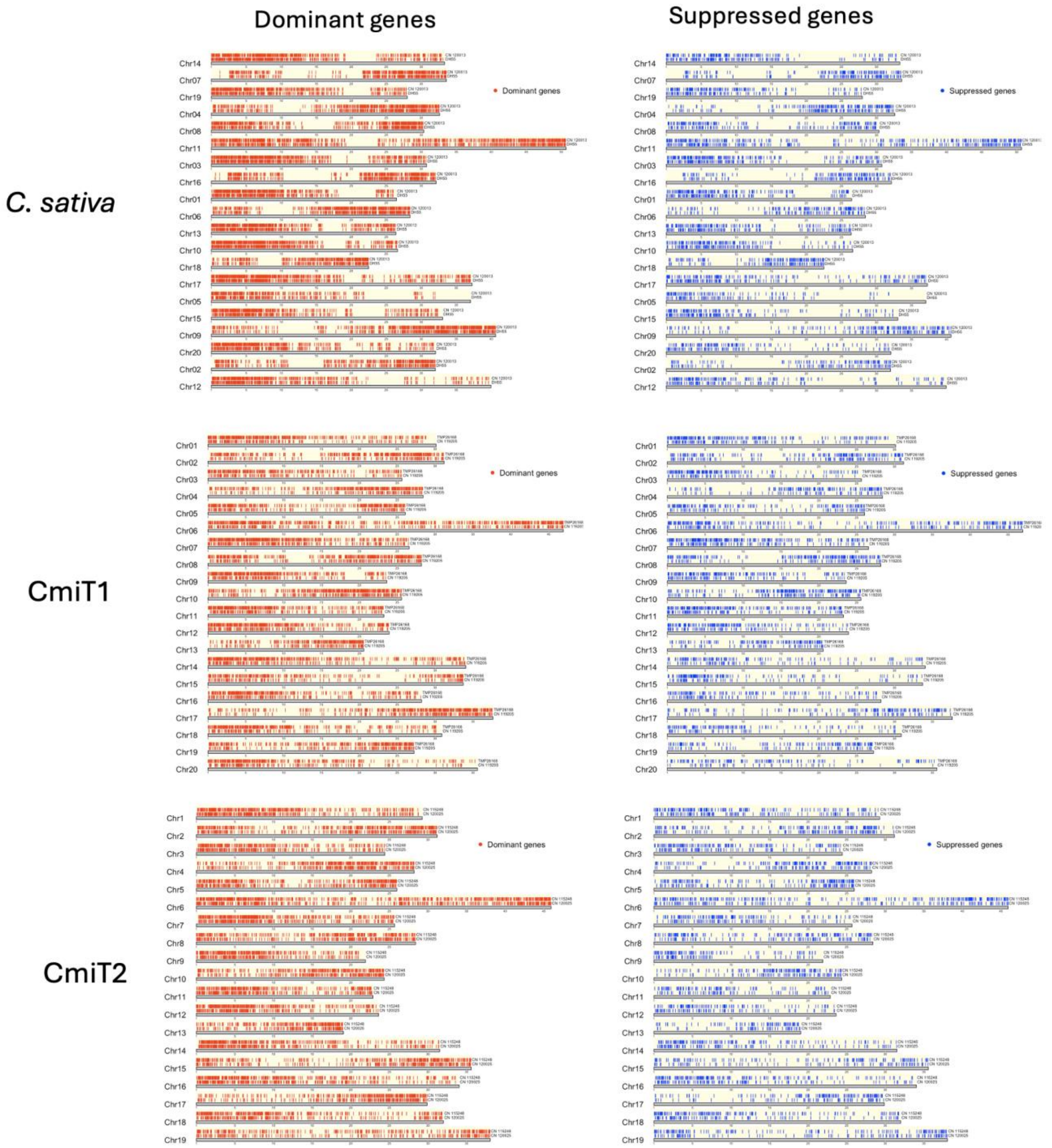

Supplementary Figure 11. **Distribution of dominant and suppressed genes in *Camelina* hexaploid genomes.** Dominant genes are shown in red while suppressed genes are shown in blue. The results for two accessions are shown in each instance.

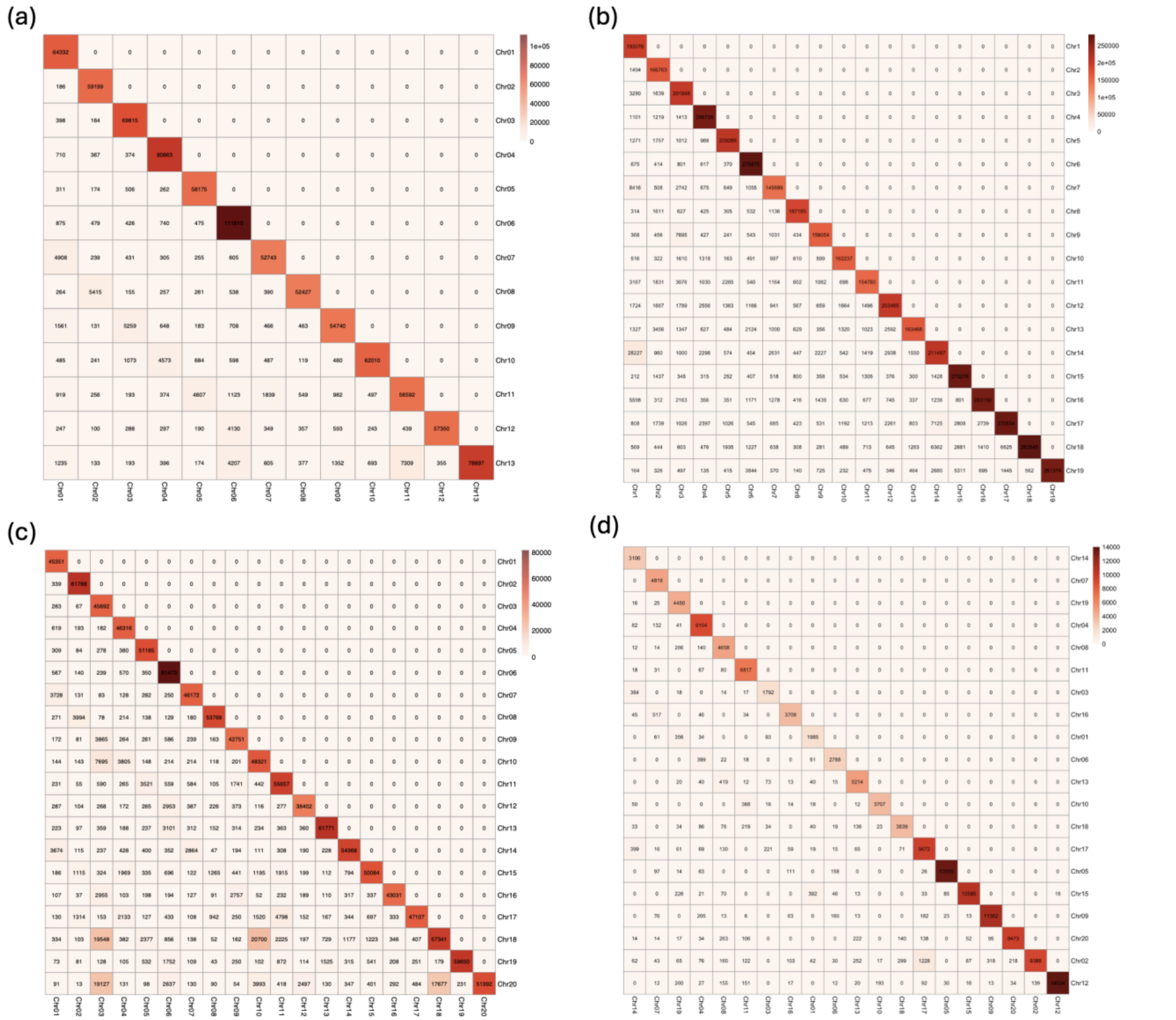

Supplementary Figure 12. **Inter- and Intra-chromosomal Interaction as explained by chromatin capture confirmation methods**. Visualization of contact points shown in Supplementary Data 11-14 for (a) CN119243, (b) CN120025, (c) CN119205, and (d) DH55 accession. CN119243 is Cmi4X, CN119205 is CmiT1, CN120025 is CmiT2, and DH55 is CsaDH55.

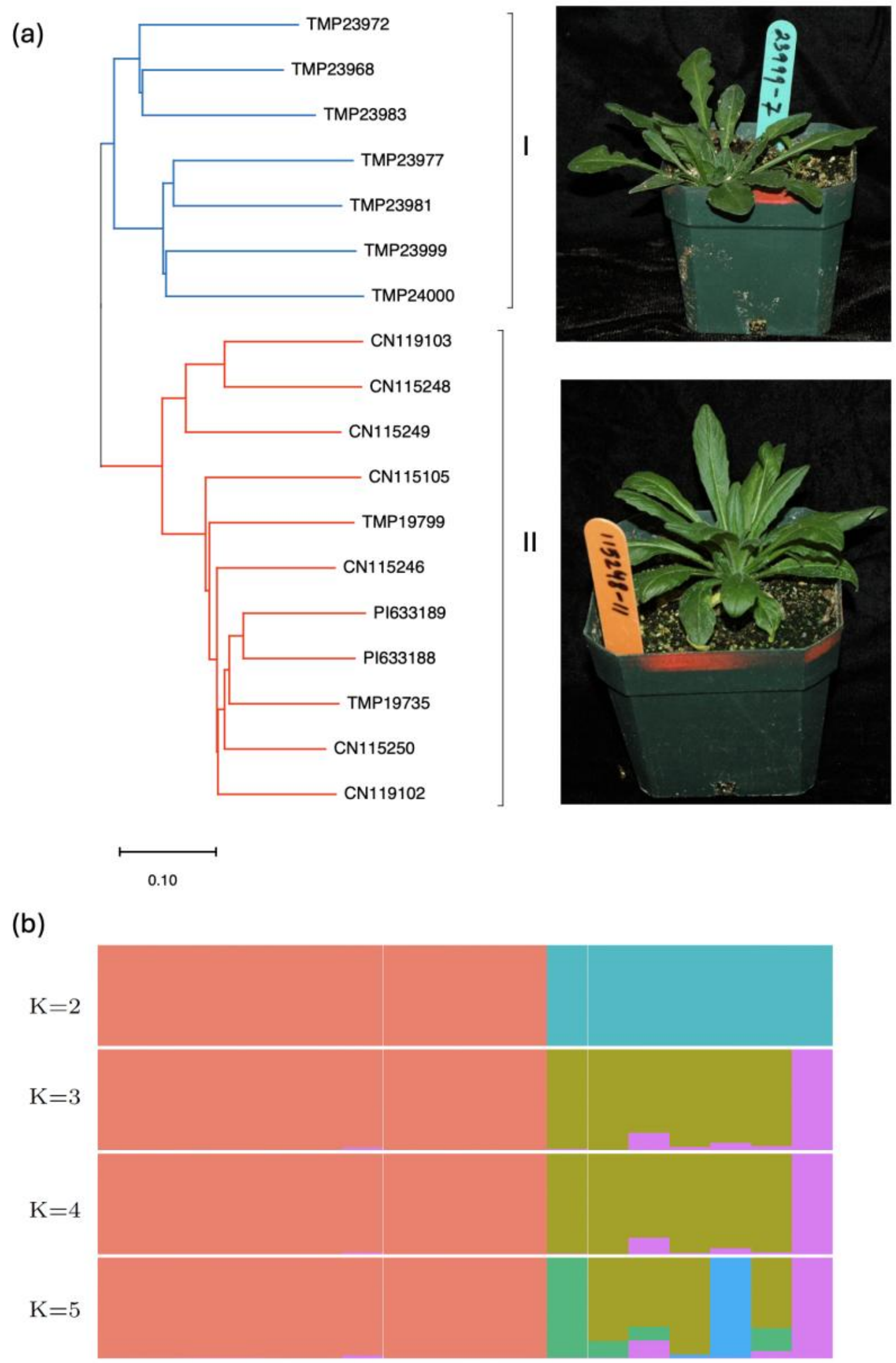

Supplementary Figure 13. **Genetic relationship among 18 genotypes from *Camelina microcarpa*.** NJ based phylogenetic tree where colour of branches represent genotypes in respective sub-populations (a), and population structure analysis with assignment probabilities (b).

(a) F_2 lines- 83-8

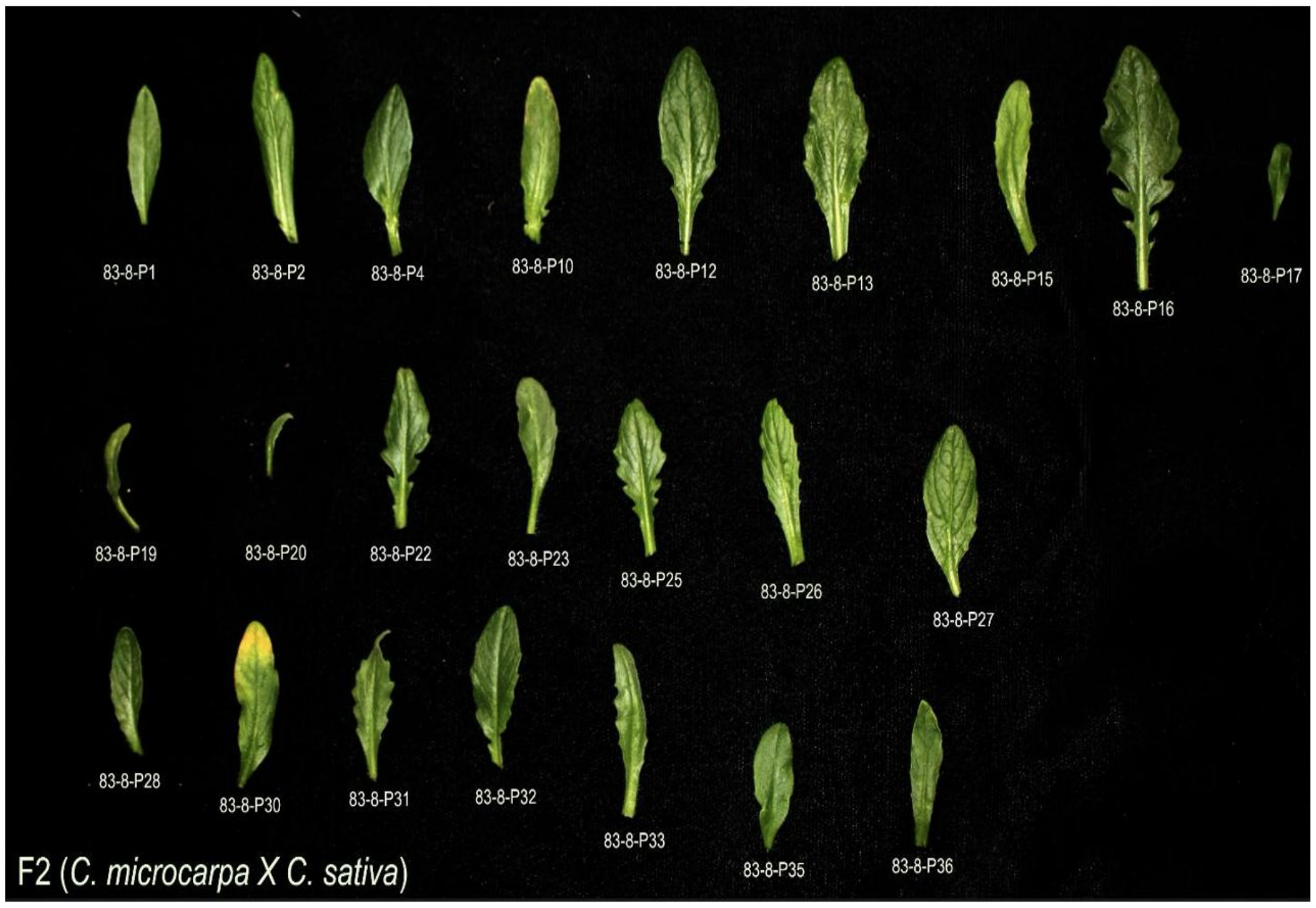

(b) F_2 lines-82-6

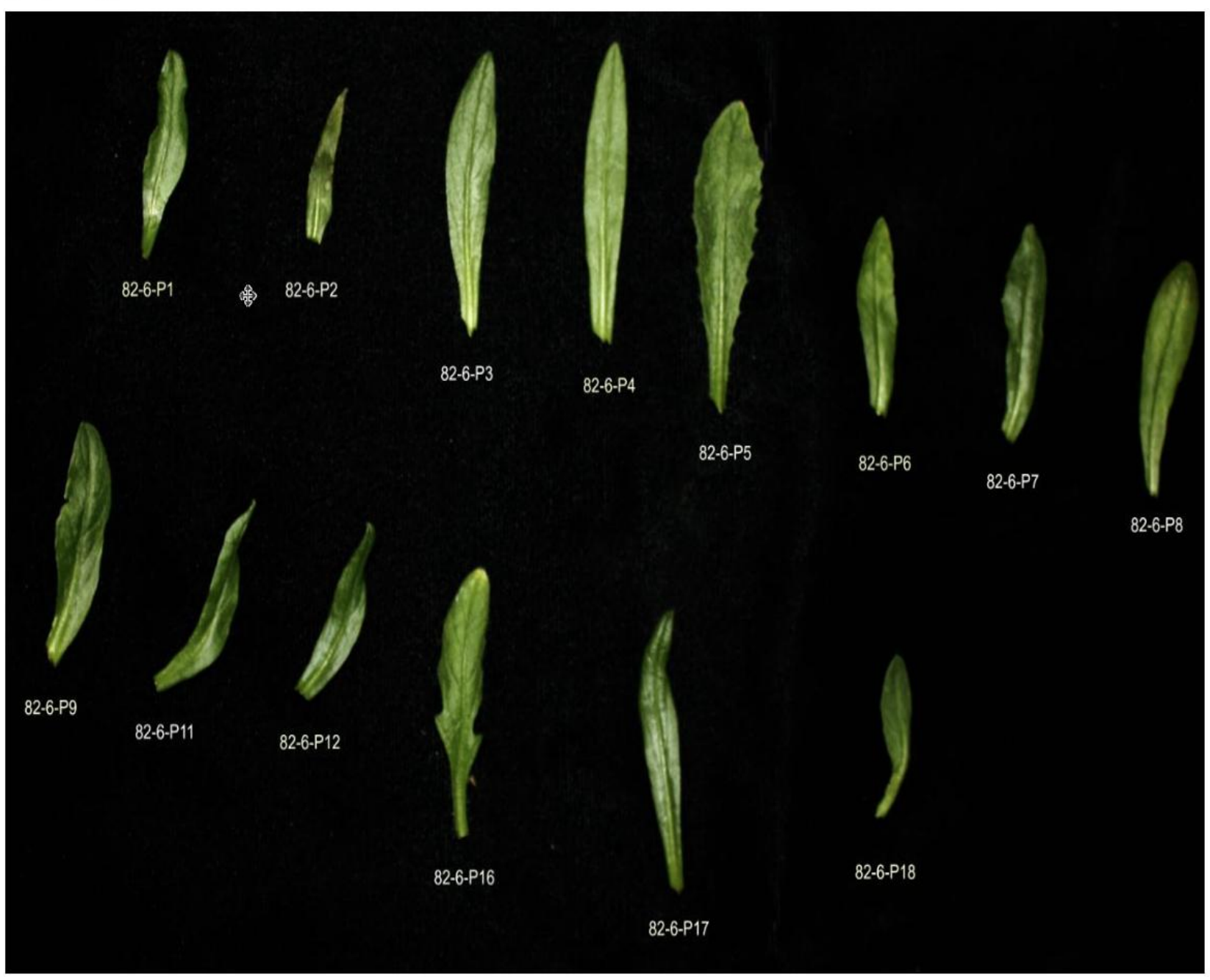

Supplementary Figure 14. **Morphological variation in F_2 lines developed from interspecific hybridization between *C. microcarpa* Type 2 and *C. sativa.*** The same parental lines were used to develop segregating lines; however, two different F_1 (a) 83-8 and (b) 82-6 were used to generate F_2 lines.

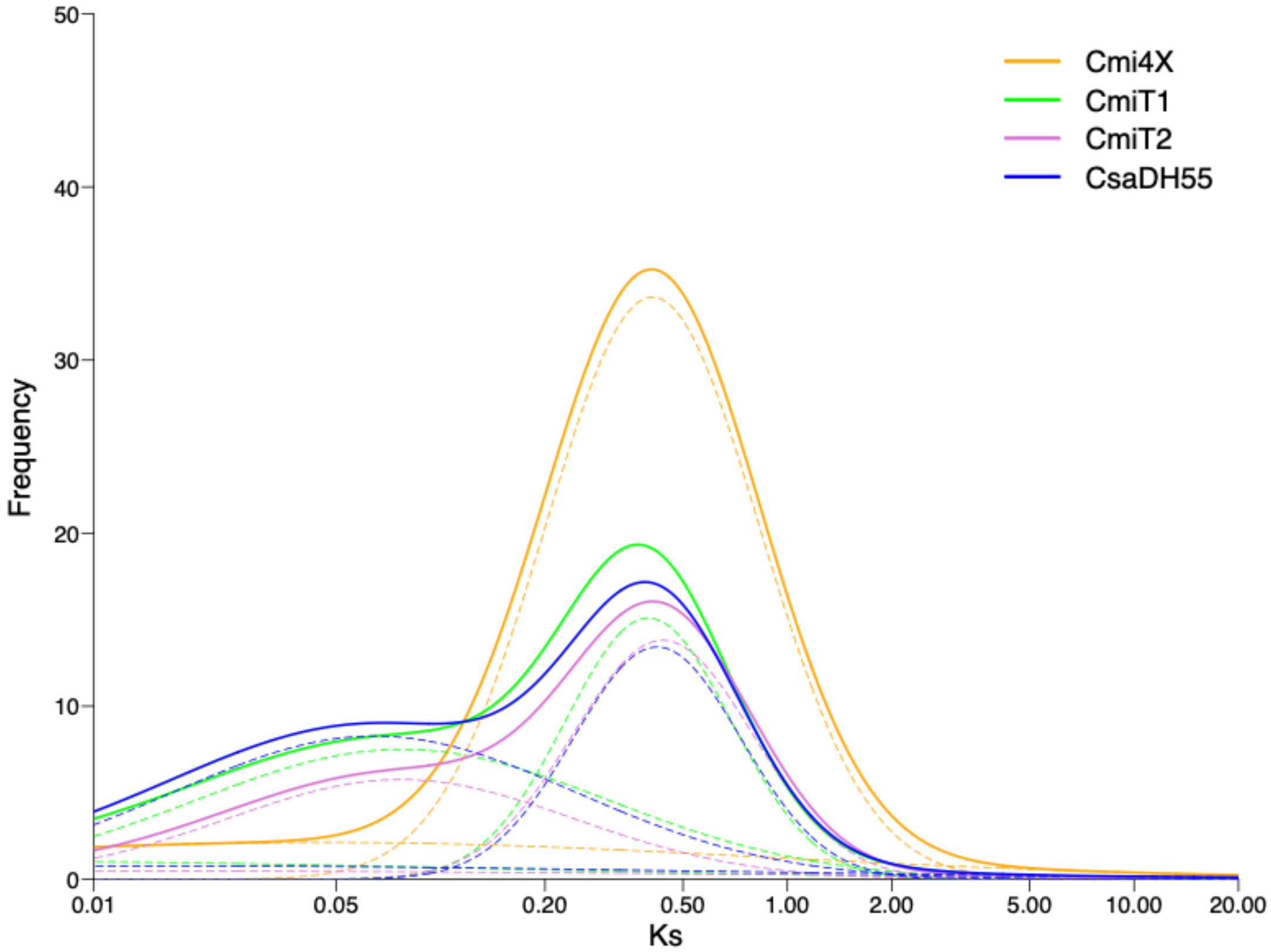

Supplementary Figure 15. **Ks analysis with tandem duplicate genes in *Camelina* species.** Dotted lines indicates different components to fit the gaussian model for the respective Ks values.

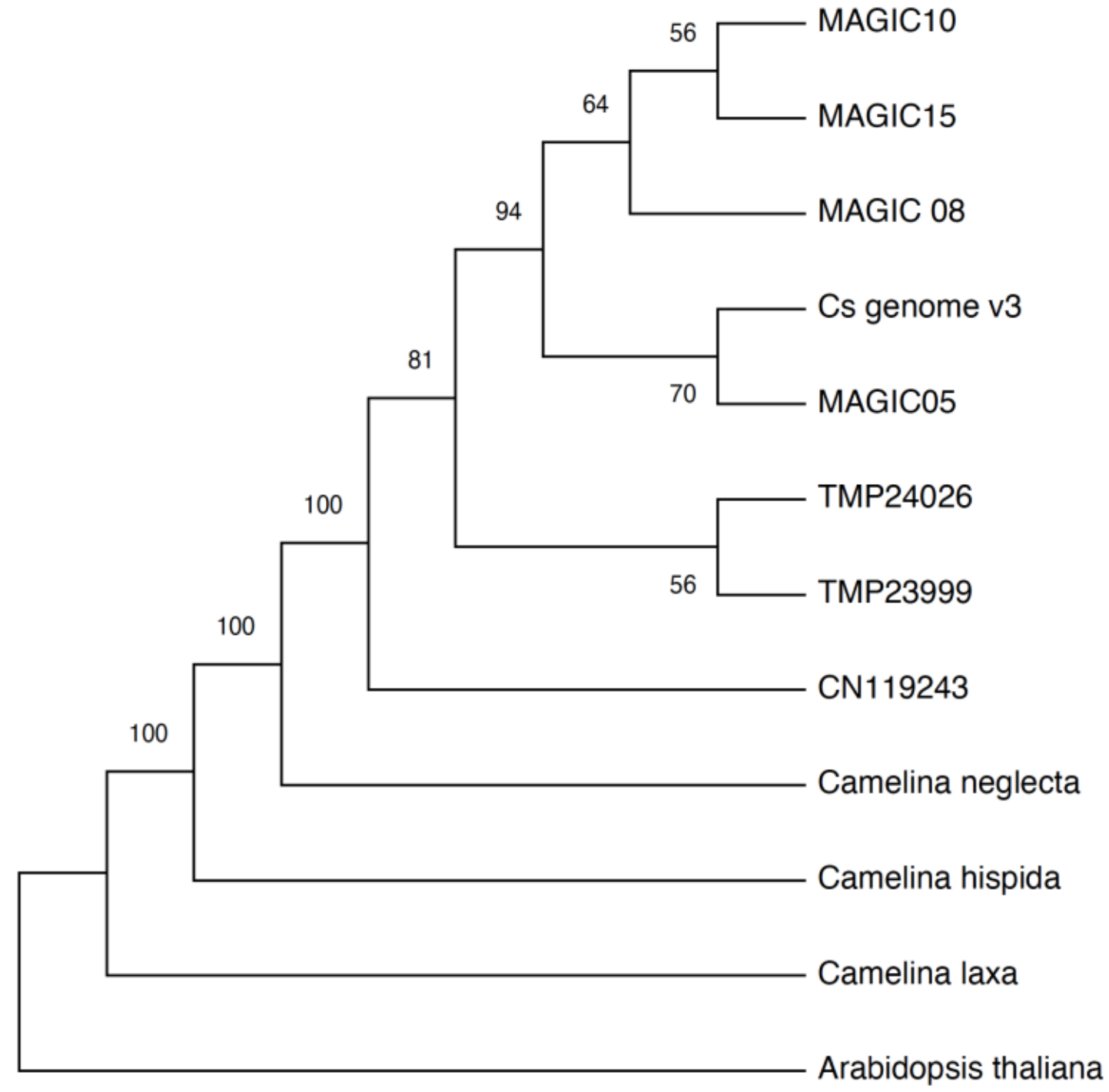

Supplementary Figure 16. Phylogenetic relationship among *Camelina* species based on whole chloroplast assembly. The *Camelina neglecta*, *C. laxa*, *C. hispida* were adopted from Martin *et al*.[2]; CN119243 (*C. microcarpa* tetraploid (n=13), TMP24026 (*C. microcarpa* T1), TMP23999 (*C. microcarpa* T2), Cs genome v3 (DH55) were generated in this study, and MAGIC05, MAGIC08, MAGIC10 and MAGIC15 were unpublished genomes of *Camelina sativa*.

Supplementary references

1. Kagale S, *et al*. The emerging biofuel crop *Camelina sativa* retains a highly undifferentiated hexaploid genome structure. *Nat. Comm.* **5**, 3706 (2014).
2. Martin SL, *et al.* Insights from the genomes of 4 diploid *Camelina* spp. *G3: Genes, Genomes, Genetics* **12**, jkac182 (2022).
