# Supplementary material for: A fourth subgenome of the Camelina genus reveals gene dominance is influenced by chromosomal proximity
Source: Nat Commun. 2026 Jun 26;17:7992. doi: 10.1038/s41467-026-74800-8 (PMC13448894; doi:10.1038/s41467-026-74800-8)
Supplement: Supplementary file 3 [file 41467_2026_74800_MOESM2_ESM.pdf]

nature portfolio

Peer Review File

A fourth subgenome of the *Camelina* genus reveals gene dominance is influenced by chromosomal proximity

Corresponding Author: Dr Isobel Parkin

This file contains all reviewer reports in order by version, followed by all author rebuttals in order by version.

Version 0:

Reviewer comments:

Reviewer #1

(Remarks to the Author)
In this manuscript, Chaudhary et al. sequence four new genomes of Camelina (a tetraploid and two hexaploidy C. macrocarpa, and a hexaploidy C sativa) and compare them to each other and to previously published Camelina genomes (e.g., Camelina neglecta, C. hispida, C. laxa, and so forth; Chaudhary et al. 2020, Mandakova et al. 2019, Martin et al. 2022).

The methods and results are sound and reproducible. The figures, tables, and results are clear and well written (including the numerous supplemental materials). There are several noteworthy results in this study.

First, Camelina macrocarpa has been of interest as a relative of domesticated Camelina sativa (Brock et al. 2018, 2019, 2022; Chaudhary et al. 2020; Mandakova et al. 2019). This study dissects a previously uncharacterized foruth subgenome found in C. macrocarpa Type 2, including its karyotype evolution (Figure 2 and hidden in Supplemental Figure 7).

Second, there is a generally well-accepted orthodoxy around "rules" for subgenome evolution, biased fractionation, and subgenome dominance in allopolyploids. However, there are some exceptions to these rules (e.g., cucurbits, soybean, tef); and this study adds another interesting example to consider when considering the various possible mechanisms that may "control" subgenome dominance.

In summary, this study provides genomic comparisons among all ploidy levels in Camelina to understand phylogenetic relationships, karyotype changes, subgenome evolution, and subgenome dominance. In addition, the study provides new insights into allopolyploid evolution with a counter-example to the "typical" two-step process seen in Brassica and other polyploid species. Finally, Camelina sativa is an important oilseed crop, and this study illuminates a path for increasing genetic and phenotypic diversity with the findings shown with C. macrocarpa Type 2, and perhaps for understanding homoeologous recombination more broadly.

Reviewer #2

(Remarks to the Author)
This manuscript describes the discovery of a novel and highly rearranged fourth subgenome in hexaploid Camelina microcarpa Type 2, challenging the established two-step model of Camelina evolution. It reveals that genome expression dominance is negatively correlated with inter-subgenome chromatin interaction frequencies, suggesting that nuclear chromosome proximity may play a key regulatory role. Successful interspecific hybridization further demonstrates the potential to harness this diversity for crop improvement. The identification of a new case of subgenome expression dominance—characterized by higher gene expression—is valuable for understanding the mechanisms underlying differential subgenome expression. However, I have several major concerns that need to be addressed before the manuscript can be accepted.

The term "subgenome dominance" typically refers to a subgenome with higher gene density, which is often associated with elevated gene expression. In this study, however, the authors use it to describe expression bias alone—more accurately termed "gene expression dominance"—which is not necessarily linked to the classical two-step evolutionary model. This distinction should be clearly defined and consistently used throughout the manuscript.

While the authors present a new subgenome in the Camelina genus and propose a novel mechanism for gene expression dominance, they do not provide sufficient detail on the genomic distribution of dominant genes. For instance, analyses showing how dominant genes are distributed across chromosomes would strengthen the claims.

Although the authors observe differences in chromatin interaction patterns—higher interactions between SG1 and SG2 in CmiT1 and CsaDH55, and between SG1 and SG3 in CmiT2—no causal evidence is provided to link these structural differences to expression dominance. Furthermore, the authors do not account for other known contributors to expression bias, such as transposable element density, which is often associated with epigenetic silencing and subgenome dominance.

The analyses of resistance genes and FLC genes, while interesting, are not directly relevant to the central theme of genome-wide expression dominance and could be moved to supplementary material or reframed to better support the main hypothesis.

Finally, I recommend revising the title for accuracy and clarity. A more appropriate title might be: "A new subgenome in the Camelina genus reveals gene expression dominance linked to chromosomal proximity."

Reviewer #3

(Remarks to the Author)
General assessment:
The manuscript by Chaudhary et al. presents high-quality genome assemblies of several Camelina species, including a tetraploid and two hexaploid C. microcarpa accessions, and the cultivated hexaploid C. sativa. The authors describe a structurally distinct third subgenome in C. microcarpa Type 2 and explore its evolutionary origin, chromatin architecture, and gene expression dominance. While the technical execution is solid and the data are extensive, I have significant concerns regarding the novelty, conceptual depth, and interpretative clarity of the study, particularly in light of prior work on Camelina genome evolution.

Major comments:
1. Novelty and conceptual contribution
The structural composition and evolutionary history of Camelina polyploids, including the identification of distinct subgenomes and their progenitors, have already been addressed in several prior studies: Mandakova et al., 2019 reconstructed the origin of diploid Camelina species, tetraploid C. rumelica, hexaploid C. sativa, and subgenome composition of the polyploids. Chaudhary et al., 2020 described the two hexaploid types of C. microcarpa and their subgenomic relationships. Martin et al., 2022 sequenced four diploid Camelina species, confirming the role of C. neglecta and C. hispida as progenitors of C. sativa. Mandaková & Lysak, 2022 proposed the tetraploid Camelina microcarpa as the maternal progenitor of C. sativa. Chaudhary et al., 2023 provided a high-quality genome of C. neglecta, identifying it as the progenitor of SG1 in hexaploid C. sativa. Blume et al., 2023 proposed an integrated evolutionary model for the genus, emphasizing the role of C. neglecta in tetra- and hexaploid formation. Brock et al., 2024 conducted a pangenome analysis of C. sativa, identifying 13 subpopulations and SG3 dominance in reproductive tissues. These studies collectively diminish the novelty of the current manuscript's evolutionary claims. The newly described SG3-T2 subgenome in C. microcarpa Type 2, while structurally divergent, does not represent a fundamentally new lineage but rather a rearranged derivative of the known C. neglecta-like genome. The term "new subgenome" may be misleading and should be clarified.

2. Subgenome dominance and chromatin architecture
The manuscript claims a negative correlation between inter-subgenome chromatin contacts and gene expression dominance. While this is conceptually interesting and consistent with findings in cotton and wheat, the analysis lacks depth:
- No epigenomic data (e.g. methylation, histone marks) are presented, despite the authors having access to such datasets.
- The dominance of SG3 in C. sativa and SG2 in C. microcarpa T2 is not sufficiently contextualized or mechanistically explained.
- RNA-seq data are derived solely from leaf tissue; cross-tissue validation (e.g. floral or root tissues) would strengthen the conclusions.
- Mapping bias in RNA-seq should be addressed, especially given the divergence among subgenomes. Reporting unique mappability per subgenome would help.

3. Gene annotation and methodological clarity
The manuscript mentions RNA-seq data and the use of Helixer for gene annotation, but the integration of these approaches is unclear. It appears that RNA-seq was used only marginally, which is a missed opportunity for improving annotation accuracy.

4. Hybridization and breeding potential
The interspecific hybridization experiments between C. sativa and C. microcarpa T2 are promising but underdeveloped:
- Fertility data are limited and not statistically analyzed.
- Segregation patterns and chromosomal behavior in F2 and BC1F2 generations are described qualitatively.
- The potential for introgression is discussed, but practical implications for breeding remain speculative.

5. RGA and FLC analysis
The analysis of resistance gene analogs appears disconnected from the main narrative. The rationale for including this section is unclear. Similarly, the structural variation in FLC is interesting but not validated (e.g. PCR, coverage plots). The

functional relevance of these findings is not demonstrated.

Technical and presentation issues
- Hi-C methodology: The use of multiple Hi-C protocols (Omni-C, Proximo) is not justified. Figures should clearly indicate which method was used.
- Supplementary Figures:
- Suppl. Fig. 2: GenomeScope plots differ from expected profiles; more explanation is needed.
- Suppl. Fig. 4: Chromosome naming is inconsistent across homologous regions.
- Suppl. Fig. 5: BUSCO results should include overall percentages.
- Figures:
- Fig. 1: Add legend for track colors.
- Fig. 3: Specify tissue type and number of biological replicates.
- Language and Formatting: The manuscript contains several spelling and formatting errors that should be corrected.

Conclusion and recommendation
While the manuscript presents technically sound data and contributes to the ongoing characterization of Camelina genome evolution, it does not meet the threshold of conceptual novelty and broad impact expected for publication in Nature Communications. The evolutionary framework, subgenome relationships, and dominance patterns largely reiterate existing models. The study would benefit from deeper mechanistic insights, improved integration of available data, and clearer interpretation of its findings.

I recommend rejection in its current form. The study may be better suited for a specialized journal focused on plant genomics or polyploid evolution.

Version 1:

Reviewer comments:

Reviewer #1

(Remarks to the Author)
This is a revision of a manuscript that I had reviewed previously as Reviewer #1. I had no issues with the original submission and still find the manuscript acceptable. I bow to the opinions of the other reviewers and editors as to whether the authors' revisions are satisfactory.

Reviewer #2

(Remarks to the Author)
The newly added analysis addresses most of the previous concerns, and I appreciate the authors' efforts in providing additional analyses. However, some concerns remain. The current manuscript still appears highly descriptive, frequently relying on vague comparative expressions such as "slightly higher number" and "relatively higher numbers" without sufficient quantitative support. I recommend that the authors further refine the manuscript structure and provide more rigorous quantitative and statistical evidence to support their conclusions more convincingly.
1. The resolution of the circos plot in Figure 1 is too low, making it difficult to assess the observations presented in the main text. I recommend improving the image resolution. In addition, a more detailed description of each track should be provided in the figure legend to facilitate interpretation.
2. The chromosome numbering in Figure 1d appears inconsistent or confusing. I suggest that the authors clarify this in the figure legend or revise the figure accordingly.
3. The description that "a proliferation of repeat elements around the centromeres of SG1 and SG2 was observed in CsaDH55, particularly for Gypsy elements and Helitrons; a similar but less pronounced pattern was also detected in CmiT1, whereas the same subgenome bias was not found in CmiT2" is somewhat general. I suggest that the authors provide a supplementary table with quantitative details to better illustrate this observation.
4. The resolution of Supplementary Figure 3 is too low and should be improved.
5. For the statement "In the tetraploid and all three hexaploids, SG2 had the lowest gene density while SG3 had the highest," no supporting quantitative data are provided. I suggest that the authors include relevant data or supplementary materials to substantiate this conclusion.
6. The statements that "the tetraploid (Cmi4X) genome contained relatively higher numbers of tandemly and proximally duplicated genes compared to the first two subgenomes in all hexaploids…" and that "there was generally a higher number of tandem and proximal duplicate copies in the third subgenome of the hexaploid Camelina species" lack sufficient quantitative support. I suggest that the authors provide a more rigorous comparative analysis with appropriate data to substantiate these observations.
7. As the authors stated, "annotation of resistance genes can often be compromised by their mis-annotation as repetitive sequences." The authors should clarify how they controlled for the potential influence of repetitive sequences on their analyses, particularly given the widespread distribution of repeats across these genomes.
8. The manuscript frequently relies on vague comparative descriptions, such as "slightly higher number" and "relatively higher numbers," without sufficient quantitative support. I recommend that the authors provide precise comparative data and, where appropriate, statistical analyses to substantiate these conclusions.

Reviewer #3

(Remarks to the Author)
The authors responded to the comments in a constructive and thorough manner, addressing both conceptual and technical concerns. They acknowledged the extensive prior work on Camelina evolution and appropriately moderated their claims by replacing the term "novel subgenome" with "fourth subgenome". On mechanistic and methodological issues, the authors expanded their analyses by generating additional ONT methylation data, assessing TE density, incorporating extra RNA-seq datasets, and re-running expression analyses with Salmon to mitigate mapping bias. These steps substantially strengthen their claims regarding gene expression dominance and chromatin architecture. They also provided clearer rationale for their annotation strategy and made appropriate editorial adjustments—removing or relocating sections on RGA and FLC, improving figure legends, and clarifying Hi-C methodology. Overall, their responses are detailed, evidence-based, and effectively improve the clarity and robustness of the manuscript.

Version 2:

Reviewer comments:

Reviewer #2

(Remarks to the Author)
The authors have satisfactorily addressed all of my concerns. I recommend that the manuscript be accepted.

Open Access This Peer Review File is licensed under a Creative Commons Attribution 4.0 International License, which permits use, sharing, adaptation, distribution and reproduction in any medium or format, as long as you give appropriate credit to the original author(s) and the source, provide a link to the Creative Commons license, and indicate if changes were made.
In cases where reviewers are anonymous, credit should be given to 'Anonymous Referee' and the source.
The images or other third party material in this Peer Review File are included in the article’s Creative Commons license, unless indicated otherwise in a credit line to the material. If material is not included in the article’s Creative Commons license and your intended use is not permitted by statutory regulation or exceeds the permitted use, you will need to obtain permission directly from the copyright holder.
To view a copy of this license, visit https://creativecommons.org/licenses/by/4.0/

We would like to thank the three reviewers for the time they have invested in providing their constructive feedback; we have taken note of all comments made and edited the manuscript accordingly. We believe that we have managed to answer each of the comments and questions raised and that the resulting changes have resulted in an improved manuscript.

In response to the reviewers comments we have added additional data, additional RNASeq analyses confirmed the unexpected gene expression dominance in SG2 of the *Camelina microcarpa* T2. We have now provided results of both methylation analyses (for which new data was generated) and transposable element distribution in the context of gene proximity. There was no correlation between either of these factors and gene expression bias.

Further, detailed responses to each of the reviewers comments can be found below. We have indicated all changes in the main text as red font.

Best wishes,

Isobel Parkin

REVIEWER COMMENTS

<u>Reviewer #1 (Remarks to the Author):</u>

In this manuscript, Chaudhary et al. sequence four new genomes of Camelina (a tetraploid and two hexaploidy C. macrocarpa, and a hexaploidy C sativa) and compare them to each other and to previously published Camelina genomes (e.g., Camelina neglecta, C. hispida, C. laxa, and so forth; Chaudhary et al. 2020, Mandakova et al. 2019, Martin et al. 2022). The methods and results are sound and reproducible. The figures, tables, and results are clear and well written (including the numerous supplemental materials). There are several noteworthy results in this study.

First, Camelina macrocarpa has been of interest as a relative of domesticated Camelina sativa (Brock et al. 2018, 2019, 2022; Chaudhary et al. 2020; Mandakova et al. 2019). This study dissects a previously uncharacterized fourth subgenome found in C. macrocarpa Type 2, including its karyotype evolution (Figure 2 and hidden in Supplemental Figure 7).

Second, there is a generally well-accepted orthodoxy around "rules" for subgenome evolution, biased fractionation, and subgenome dominance in allopolyploids. However, there are some exceptions to these rules (e.g., cucurbits, soybean, tef); and this study adds another interesting example to consider when considering the various possible mechanisms that may "control" subgenome dominance.

In summary, this study provides genomic comparisons among all ploidy levels in Camelina to understand phylogenetic relationships, karyotype changes, subgenome evolution, and subgenome dominance. In addition, the study provides new insights into allopolyploid evolution with a counter-example to the "typical" two-step process seen in Brassica and other polyploid species. Finally, Camelina sativa is an important oilseed crop, and this study illuminates a path for increasing genetic and phenotypic diversity with the findings shown with C. macrocarpa Type 2, and perhaps for understanding homoeologous recombination more broadly.

Response: We would like to thank reviewer 1 for their considerate review of our work and their positive impression of these findings in the context of past and future work.

<u>Reviewer #2 (Remarks to the Author):</u>

This manuscript describes the discovery of a novel and highly rearranged fourth subgenome in hexaploid Camelina microcarpa Type 2, challenging the established two-step model of Camelina evolution. It reveals that genome expression dominance is negatively correlated with inter-subgenome chromatin interaction frequencies, suggesting that nuclear chromosome proximity may play a key regulatory role. Successful interspecific hybridization further demonstrates the potential to harness this diversity for crop improvement. The identification of a new case of subgenome expression dominance—characterized by higher gene expression—is valuable for understanding the mechanisms underlying differential subgenome expression. However, I have several major concerns that need to be addressed before the manuscript can be accepted.

Response: Thank you for the positive comments and we hope that we have addressed your concerns below.

The term "subgenome dominance" typically refers to a subgenome with higher gene density, which is often associated with elevated gene expression. In this study, however, the authors use it to describe expression bias alone—more accurately termed "gene expression dominance"—which is not necessarily linked to the classical two-step evolutionary model. This distinction should be clearly defined and consistently used throughout the manuscript.

Response: We would agree that this more nuanced description of "gene expression dominance" rather than the all-encompassing "genome dominance" is more appropriate in the context of polyploid genomes that have yet to experience, or perhaps would never experience, substantial gene fractionation. In the introduction we had indicated that genome dominance has been assessed in the context of gene expression "genes commonly show evidence of higher gene

expression for a particular subgenome (genome dominance)". We have reassessed the text in this light and added where necessary 'gene expression' when referring to dominance.

While the authors present a new subgenome in the Camelina genus and propose a novel mechanism for gene expression dominance, they do not provide sufficient detail on the genomic distribution of dominant genes. For instance, analyses showing how dominant genes are distributed across chromosomes would strengthen the claims. Although the authors observe differences in chromatin interaction patterns—higher interactions between SG1 and SG2 in CmiT1 and CsaDH55, and between SG1 and SG3 in CmiT2—no causal evidence is provided to link these structural differences to expression dominance. Furthermore, the authors do not account for other known contributors to expression bias, such as transposable element density, which is often associated with epigenetic silencing and subgenome dominance.

Response: We have now provided information on the distribution of dominant genes across the genomes (Supplemental Figure 11). Although at the whole genome level differences in gene expression dominance among thousands of genes are quite difficult to observe.

The analyses of transposable element density and CpG methylation status proximal to the genes is easier to visual, and compare across subgenomes in a meaningful manner, and we agree that this information is highly relevant to gene expression dominance. We have now provided this information for each of the hexaploid species in the main text, including Figure 4.

The analyses of resistance genes and FLC genes, while interesting, are not directly relevant to the central theme of genome-wide expression dominance and could be moved to supplementary material or reframed to better support the main hypothesis.

Reponse: We agree these analyses may not be as relevant to the main theme of the manuscript. However, we have noticed, as others, that annotation of RGA sequences is often a good additional metric for assessing the quality of the annotation, so we have included this information in the annotation section, but moved the figure to the supplementary data. We have removed the FLC analyses.

Finally, I recommend revising the title for accuracy and clarity. A more appropriate title might be: "A new subgenome in the Camelina genus reveals gene expression dominance linked to chromosomal proximity."

Response: We have changed the title accordingly.

Reviewer #3 (Remarks to the Author):

Major comments:

1. Novelty and conceptual contribution

The structural composition and evolutionary history of Camelina polyploids, including the identification of distinct subgenomes and their progenitors, have already been addressed in several prior studies: Mandakova et al., 2019 reconstructed the origin of diploid Camelina species, tetraploid C. rumelica, hexaploid C. sativa, and subgenome composition of the polyploids. Chaudhary et al., 2020 described the two hexaploid types of C. microcarpa and their subgenomic relationships. Martin et al., 2022 sequenced four diploid Camelina species, confirming the role of C. neglecta and C. hispida as progenitors of C. sativa. Mandaková & Lysak, 2022 proposed the tetraploid Camelina microcarpa as the maternal progenitor of C. sativa. Chaudhary et al., 2023 provided a high-quality genome of C. neglecta, identifying it as the progenitor of SG1 in hexaploid C. sativa. Blume et al., 2023 proposed an integrated evolutionary model for the genus, emphasizing the role of C. neglecta in tetra- and hexaploid formation. Brock et al., 2024 conducted a pangenome analysis of C. sativa, identifying 13 subpopulations and SG3 dominance in reproductive tissues. These studies collectively diminish the novelty of the current manuscript's evolutionary claims. The newly described SG3-T2 subgenome in C. microcarpa Type 2, while structurally divergent, does not represent a fundamentally new lineage but rather a rearranged derivative of the known C. neglecta-like genome. The term "new subgenome" may be misleading and should be clarified.

Response: We agree that there have been remarkable strides in determining the evolutionary history of *Camelina* species, and we have been careful to reference all the relevant studies mentioned above. Although we had previously determined in Chaudhary et al (2020) that there were two potential hexaploid species of *C. microcarpa,* and noted the homology of the new SG3' to SG1, or the SG most similar to *C. neglecta*, we still did not understand the structure of this genome, and since no extant relatives of the fourth subgenome have yet been found, it is difficult to determine the exact origin of the subgenome. We would argue that the fourth subgenome has value in its own right, similar to what has been observed in other *Brassicaceae* species, for example although *Brassica rapa* and *Brassica oleracea* are derived from the same lineage they are unique species, with intrinsic value and stories to tell. The new assemblies provide an excellent foundation for anyone studying polypoidy and those attempting to introduce novel variation into the platform oilseed *C. sativa* from the wild relatives. However, in consideration of the reviewer's comment we have changed the wording of 'novel subgenome' to 'fourth subgenome'.

2. Subgenome dominance and chromatin architecture

The manuscript claims a negative correlation between inter-subgenome chromatin contacts and

gene expression dominance. While this is conceptually interesting and consistent with findings in cotton and wheat, the analysis lacks depth:

- No epigenomic data (e.g. methylation, histone marks) are presented, despite the authors having access to such datasets.

- The dominance of SG3 in C. sativa and SG2 in C. microcarpa T2 is not sufficiently contextualized or mechanistically explained.

- RNA-seq data are derived solely from leaf tissue; cross-tissue validation (e.g. floral or root tissues) would strengthen the conclusions.

- Mapping bias in RNA-seq should be addressed, especially given the divergence among subgenomes. Reporting unique mappability per subgenome would help.

Response: Although we did have long read data from all genomes we did not have sufficient data of the appropriate type for all the species to accurately call the methylation patterns across all the genomes. We have rectified this by generating additional ONT data for the *C. microcarpa* T2 genome. This has allowed us to contextualize the CpG methylation status in the context of the genes and in addition we have studied the distribution of the transposable elements to assess their potential impact on gene expression in the different subgenomes.

For *Camelina sativa* we already had analysed and presented RNASeq data from 12 tissue types, now clearly indicated in the figure legend. We have now added additional RNASeq data from all hexaploid species from etiolated seedling tissue to our RNASeq analyses, which confirms the dominant gene expression pattern of SG2 in *C. microcarpa* T2. We understand the reviewers concern regarding mapping bias, so we have now extracted unique mapping rates across the three subgenomes for the new data in the supplementary data; multi-mapping ranged from 13 to 18%. In addition, since k-mer based pseudo-alignment programs, such as Salmon and Kallisto, have been shown to be more specific when carrying out read alignment in polyploid species, we have re-run the analyses of the data using Salmon, which confirmed the original results.

We have now provided additional analyses of the transposable element distribution and CpG methylation status, to show they do not correlate with expression dominance. The mechanism for genome expression bias as suggested in the text is inferred to relate to accessibility of chromatin, the physical organization of which provides the optimal environment for gene expression in the more 'open' subgenome.

3. Gene annotation and methodological clarity

The manuscript mentions RNA-seq data and the use of Helixer for gene annotation, but the integration of these approaches is unclear. It appears that RNA-seq was used only marginally,

which is a missed opportunity for improving annotation accuracy.

Response: Helixer has provided to be very effective at annotating Brassicaceae genomes, probably due to the significant training data available for high quality genomes from this family. We could have perhaps provided additional alternative transcript annotation for the genomes based on our RNASeq data, but we were not convinced this would constitute a significant improvement of the current annotation. Moving forward the most optimal mechanism to actually improve genome annotations would be with either significant human curation or the addition of substantial long read RNASeq data, which may be possible as these technologies improve.

4. Hybridization and breeding potential
The interspecific hybridization experiments between C. sativa and C. microcarpa T2 are promising but underdeveloped:
- Fertility data are limited and not statistically analyzed.
- Segregation patterns and chromosomal behavior in F2 and BC1F2 generations are described qualitatively.
- The potential for introgression is discussed, but practical implications for breeding remain speculative.

Response: The intent was to show the possible value of the *C. microcarpa* genomes for capturing traits in *C. sativa*, which has not previously been shown. The fertility data is not in of itself that meaningful, since it purely indicates issues of chromosomal imbalance, which was to be expected. The data provided focuses on the potential for the use of inter-specific crosses between *C. sativa* and *C. microcarpa* T2; showing molecular genetic data that indicates exchange between the genomes has occurred, which is novel and has not previously been shown and as the reviewer suggests is promising for extracting value from the wild *Camelina* species.

5. RGA and FLC analysis
The analysis of resistance gene analogs appears disconnected from the main narrative. The rationale for including this section is unclear. Similarly, the structural variation in FLC is interesting but not validated (e.g. PCR, coverage plots). The functional relevance of these findings is not demonstrated.

Response: We have either moved or removed these sections.

Technical and presentation issues

- Hi-C methodology: The use of multiple Hi-C protocols (Omni-C, Proximo) is not justified. Figures should clearly indicate which method was used.

Response: The use of the different methodologies merely reflects changes in technologies over the span of the project. However, the methods are somewhat different, Omni-C, since there is no restriction enzyme bias, provides a better resolution across the whole genome. Thus we wanted to ensure that the use of the two technologies had not influenced the distribution of the HiC contacts found and we compared the two methods. We have indicated in the figure legend which method was used to generate the data.

- *Supplementary Figures:*

- Suppl. Fig. 2: GenomeScope plots differ from expected profiles; more explanation is needed.

Response: The GenomeScope plots are as expected for diploids. Additional peaks would only been observed if there were high levels of heterozygosity. It is possible to detect ploidy levels using Smudgeplot but that seemed unnecessary in the context of whole genome assemblies.

- Suppl. Fig. 4: Chromosome naming is inconsistent across homologous regions.

The chromosome naming was different as we have followed original nomenclature for *C. sativa*, whereas we have used chromosome nomenclature for *C. microcarpa* to reflect the three subgenomes. It would be preferable to change the chromosome names for *C. sativa*; however, we have found with other species that such changes can cause confusion within the community.

- Suppl. Fig. 5: BUSCO results should include overall percentages.

Response: The overall BUSCO scores are shown in Table 1 also we have updated supplementary Figure 5 to include the overall percentages.

- Figures:

- Fig. 1: Add legend for track colors.

- Fig. 3: Specify tissue type and number of biological replicates.

Response: We have updated the figure legends

Response to Reviewer #2:

The newly added analysis addresses most of the previous concerns, and I appreciate the authors' efforts in providing additional analyses. However, some concerns remain. The current manuscript still appears highly descriptive, frequently relying on vague comparative expressions such as "slightly higher number" and "relatively higher numbers" without sufficient quantitative support. I recommend that the authors further refine the manuscript structure and provide more rigorous quantitative and statistical evidence to support their conclusions more convincingly.

Response: We thank the reviewer for their further suggestions for refinements. We understand the need to be specific and have added further qualifications for those statements that the reviewer noted below. We have also removed where unnecessary qualifying adverbs such as 'slightly'. Any changes to the text have been noted in red.

The resolution of the circos plot in Figure 1 is too low, making it difficult to assess the observations presented in the main text. I recommend improving the image resolution. In addition, a more detailed description of each track should be provided in the figure legend to facilitate interpretation.

Response: Pasting the figure into the word document does affect the resolution, the figure has now been added to the word file with no compression which hopefully solve this issue. We have also added a more detailed description of each track in the updated Figure legend, see below.

Figure 1. Genome of *Camelina* species.

(a) *Camelina microcarpa* tetraploid Cmi4X, (b) *C. microcarpa* CmiT1, (c) *C. microcarpa* CmiT2, and (d) *C. sativa* CsaDH55. The outer track (A) represents chromosomes, with the subgenomes coloured in blue, orange, and green and the centromeres indicated by a black band. The remaining tracks from the outer to inner circle represent gene density (1 to 50 per 100 kb bin) (B), gene expression (log(FPKM+1) (C), distribution per 100 kb bin of repeat elements (1 to 908) (D), gypsy elements (1 to 619) (E), copia elements (1 to 69) (F), Helitrons (1 to 600) (G), mutator elements (1 to 889) (H), and full-length LTR elements (1 to 7) (I). Note: For all *C. microcarpa* genomes the chromosomes are numbered from 1-13, 19 or 20; however, the *C. sativa* chromosomes are numbered according to the original reference genome publication as in Kagale at al. (2014), but are ordered according to their closest homologue in *C. microcarpa*.

The chromosome numbering in Figure 1d appears inconsistent or confusing. I suggest that the authors clarify this in the figure legend or revise the figure accordingly.

Response: As we explained previously we have used the same chromosome assignment for CsaDH55 as for the original publication of the short-read version of the reference genome of *C. sativa* DH55 (https://doi.org/10.1038/ncomms4706). Since the original genome has been referenced many times it would cause confusion to attempt to rename the chromosomes now. We have added this information to the figure legend (see previous response).

The description that "a proliferation of repeat elements around the centromeres of SG1 and SG2 was observed in CsaDH55, particularly for Gypsy elements and Helitrons; a similar but less pronounced pattern was also detected in CmiT1, whereas the same subgenome bias was not found in CmiT2" is somewhat general. I suggest that the authors provide a supplementary table with quantitative details to better illustrate this observation.

Response: We have provided a new supplementary Table S4c: Density (average per 100 kb bin) of major repeat elements across centromeric (cent) and non-centromeric (non-cent) regions.

Element	Subgenome	Cmi4X		CmiT2		CmiT1		CsaDH55	
		Cent*	non-cent	Cent	non-cent	Cent	non-cent	Cent	non-cent
Gypsy	SG1	16.2	10.1	25.7	11.2	16.3	10.0	207.8	19.2
	SG2	19.8	8.7	29.6	9.4	18.6	8.9	173.8	13.0
	SG3			25.8	13.1	17.2	11.6	46.0	12.1
Copia	SG1	5.0	4.4	6.8	4.5	4.8	4.9	12.1	5.4
	SG2	7.5	4.0	5.6	4.1	8.1	4.6	10.3	4.9
	SG3			5.4	4.4	5.9	4.6	13.8	5.4
Helitron	SG1	14.5	23.2	30.5	27.3	146.5	31.4	185.1	35.2
	SG2	7.7	21.9	33.1	25.3	139.1	29.5	192.6	35.2
	SG3			37.5	29.3	34.5	34.3	28.6	33.0
Mutator	SG1	37.4	4.2	5.9	4.4	79.5	4.3	1.1	3.9
	SG2	69.5	3.8	5.2	4.2	72.5	4.3	1.0	3.9
	SG3			5.8	4.5	17.4	4.7	1.2	5.9

The resolution of Supplementary Figure 3 is too low and should be improved.

Response: The figure has been updated.

For the statement "In the tetraploid and all three hexaploids, SG2 had the lowest gene density while SG3 had the highest," no supporting quantitative data are provided. I suggest that the authors include relevant data or supplementary materials to substantiate this conclusion.

Response: We had provided the gene numbers across the different genomes and chromosomes in Supplementary Table 6; however, the reviewer has drawn attention to an oversight on our part. We had used gene density in place of gene number, where SG2 has the lowest gene number, yet it actually has the highest gene density due to the smaller genome size of SG2. The opposite is also true for SG3 which has the highest number of genes but is the largest. We have added the gene density (gene/kb) to Supplementary Table 6 and updated the text.

In the tetraploid and all three hexaploids, SG2 had the lowest gene number while SG3 had the highest, yet due to the subgenome size this equated to SG2 having the highest gene density while SG3 had the lowest (**Supplementary Table 6**).

The statements that "the tetraploid (Cmi4X) genome contained relatively higher numbers of tandemly and proximally duplicated genes compared to the first two subgenomes in all hexaploids…" and that "there was generally a higher number of tandem and proximal duplicate copies in the third subgenome of the 3exaploidy Camelina species" lack sufficient quantitative support. I suggest that the authors provide a more rigorous comparative analysis with appropriate data to substantiate these observations.

Response: We had provided the supporting data in Supplementary table S7. However, we had not included the total numbers for each sub-genome, which we have now done and this makes the statement much clearer. We have also added qualifying statements to the text and removed 'relatively' and 'generally':

The tetraploid (Cmi4X) genome contained higher numbers of tandemly and proximally duplicated genes, from 2.4-3.8 fold and 1.6-2.3 fold greater, respectively, compared to the first two subgenomes in all hexaploids ….. (Supplementary Table 7).

There was a higher number of tandem and proximal duplicate copies in the third subgenome of the hexaploid *Camelina* species, compared to SG1 and SG2, ranging from a 1.35 to 4 fold greater number (**Supplementary Table 7**).

As the authors stated, "annotation of resistance genes can often be compromised by their mis-annotation as repetitive sequences." The authors should clarify how they controlled for the potential influence of repetitive sequences on their analyses, particularly given the widespread distribution of repeats across these genomes.

Response: The annotation pipeline was run on the genome without repeat masking, which can confound the annotation of resistance genes. We have now indicated this in the methods.
