# Supplementary material for: A fourth subgenome of the Camelina genus reveals gene dominance is influenced by chromosomal proximity
Source: Nat Commun. 2026 Jun 26;17:7992. doi: 10.1038/s41467-026-74800-8 (PMC13448894; doi:10.1038/s41467-026-74800-8)
Supplement: Supplementary file 4 [file 41467_2026_74800_MOESM3_ESM.pdf]

Description of Additional Supplementary Files

File Name: Supplementary Data 1
Description: Distribution of repeat elements in the assembled genomes of *Camelina* species

File Name: Supplementary Data 2
Description:
a) Distribution of full-length LTR elements in different subgenomes of *Camelina* species
b) Distribution of full-length LTR Copia and Gypsy element families in different subgenomes of *Camelina* species (note: elements in the unanchored scaffolds are not represented)
c) Density (average per 100 kb bin) of major repeat elements across centromeric (cent) and non-centromeric (non-cent) regions. Coordinates for centromeric regions provided in Supplementary Table 3.

File Name: Supplementary Data 3
Description: Distribution of annotated genes across different subgenomes in different species of *Camelina*.

File Name: Supplementary Data 4
Description:
a) Number of duplicate genes in the assembled genomes
b) Distribution of tandem and proximal duplicate genes in assembled genomes

File Name: Supplementary Data 5
Description: Distribution of RGA in *Camelina* species

File Name: Supplementary Data 6
Description: Analysis of orthogroups in different species of *Camelina* and their subgenomes

File Name: Supplementary Data 7
Description: Synteny table of different species of *Camelina* relative to *A. thaliana*

File Name: Supplementary Data 8
Description: Number of genes showing dominant expression across subgenomes in *Camelina sativa* (n=20 chromosomes), *C. microcarpa* T1 (n=20), and *C. microcarpa* T2 (n=19 chromosomes) genome

File Name: Supplementary Data 9
Description: Genome-wide and subgenome-specific DNA methylation levels in *Camelina* accessions estimated using different long-read sequencing technologies (>=10 reads and >=50% modified).

File Name: Supplementary Data 10
Description: Methylation (CpG) percentage across different transposable elements in *Camelina* species and subgenomes.

File Name: Supplementary Data 11
Description: Identification of significant inter- and intra-chromosomal interaction as a frequency of contact read pairs in Cmi4X (CN119243). P-value calculated by FitHiC using two-sided binomial testing with correction for multiple testing to calculate q-value.

File Name: Supplementary Data 12
Description: Identification of significant inter- and intra-chromosomal interaction as a frequency of contact read pairs in CmiT2 (CN120025). P-value calculated by FitHiC using two-sided binomial testing with correction for multiple testing to calculate q-value.

File Name: Supplementary Data 13
Description: Identification of significant inter- and intra-chromosomal interaction as a frequency of contact read pairs in CmiT1 (CN119205). P-value calculated by FitHiC using two-sided binomial testing with correction for multiple testing to calculate q-value.

File Name: Supplementary Data 14
Description: Identification of significant inter- and intra-chromosomal interaction as a frequency of contact read pairs in Cs_genome_v3 (CsaDH55). P-value calculated by FitHiC using two-sided binomial testing with correction for multiple testing to calculate q-value.

File Name: Supplementary Data 15
Description: Morphological characteristics of F2 lines developed from *C. microcarpa* x *C. sativa* (CN119102 x CN120019)

File Name: Supplementary Data 16
Description: Unique read mapping rates based on STAR alignments of etiolated seedling RNASeq data
